# RNA-Interference Knockdown of *Drosophila* Pigment Dispersing Factor in Neuronal Subsets: The Anatomical Basis of a Neuropeptide's Circadian Functions

**DOI:** 10.1371/journal.pone.0008298

**Published:** 2009-12-14

**Authors:** Orie T. Shafer, Paul H. Taghert

**Affiliations:** Department of Anatomy and Neurobiology, Washington University Medical School, St. Louis, Missouri, United States of America; Yale School of Medicine, United States of America

## Abstract

**Background:**

In animals, neuropeptide signaling is an important component of circadian timekeeping. The neuropeptide pigment dispersing factor (PDF) is required for several aspects of circadian activity rhythms in *Drosophila*.

**Methodology/Principal Findings:**

Here we investigate the anatomical basis for PDF's various circadian functions by targeted PDF RNA-interference in specific classes of *Drosophila* neuron. We demonstrate that PDF is required in the ventro-lateral neurons (vLNs) of the central brain and not in the abdominal ganglion for normal activity rhythms. Differential knockdown of PDF in the large or small vLNs indicates that PDF from the small vLNs is likely responsible for the maintenance of free-running activity rhythms and that PDF is not required in the large vLNs for normal behavior. PDF's role in setting the period of free-running activity rhythms and the proper timing of evening activity under light:dark cycles emanates from both subtypes of vLN, since PDF in either class of vLN was sufficient for these aspects of behavior.

**Conclusions/Significance:**

These results reveal the neuroanatomical basis PDF's various circadian functions and refine our understanding of the clock neuron circuitry of *Drosophila*.

## Introduction

The predictable daily modulation of physiological processes is a ubiquitous characteristic of the living world. Endogenous circadian clocks drive these daily changes and are composed of cell-autonomous intracellular feedback loops of gene expression [1]. In animals, the cellular clocks that control daily activity rhythms reside in neurons located in discrete regions of the central nervous system [2]. For example, the suprachiasmatic nuclei (SCN) of mammals house the neuronal circadian clocks required for the maintenance endogenous activity rhythms (e.g., [3], [4]). Despite the seemingly cell-autonomous nature of neuronal circadian clocks [5], it is clear that the orchestration of daily behavioral rhythms depends on the network properties of heterogeneous clock-containing neurons (reviewed in [2]). Understanding how the timekeeping of molecular clocks is employed by networks of clock neurons to orchestrate rhythmic behavior is a central problem in chronobiology.

Work in several animal species has shown that neuropeptide signaling is a critical component of neuronal clock networks (Reviewed in [6] and [7]). For example, the neuropeptide vasoactive intestinal peptide (VIP) and its G-protein-coupled receptor VPAC_2_ are required for normal locomotor rhythms under constant conditions and for the maintenance of rhythmicity and synchrony among SCN neurons [8], [9].

The fly *Drosophila melanogaster* has been a fruitful model for the ways in which networks of clock-gene-expressing neurons interact to orchestrate the timing of daily activity. In this fly, approximately 150 neurons – and even more glia – express a well-characterized molecular clockwork [10], [11]. (In the present study we refer to clock-gene-expressing neurons as “clock neurons.”) Identified anatomical subsets of clock neurons have been hypothesized to serve as oscillators dedicated to discreet portions of *Drosophila*'s daily crepuscular activity rhythm [12], [13], [14], [15]. Sixteen of these clock neurons - eight large ventral-lateral neurons (l-vLNs) and eight small ventro-lateral neurons (s-vLNs) - express the neuropeptide Pigment Dispersing Factor (PDF), which is required for several aspects of locomotor rhythms [16], [17]. *Pdf^01^*mutants lack PDF peptide and show high levels of arhythmicity under constant darkness (DD) and temperature. The *Pdf^01^* flies that are rhythmic have a significantly shortened free-running period [17]. Under Light/Dark (LD) cycles consisting of 12 h of light and 12 h of darkness (LD 12∶12), *Pdf^01^* mutants fail to display the normal anticipation of the morning lights-on transition and have a significantly advanced evening peak of activity, which results in an increase in daytime activity [17]. PDF is also required for the normal adjustment of locomotor rhythms to changing day-length [18].

PDF's receptor (PDFr) has been identified and has high sequence homology to VPAC_2_ [19], [20], [21]. Most *Drosophila* clock neuron classes are receptive to PDF [22], which is thought to coordinate the normal phasing and amplitude of clock neuron molecular timekeeping [23], [18]. In addition to its roles in circadian timekeeping, PDF has been implicated as a modulator of geotactic drive [24] and sleep and arousal [25], [26], [27].

It is remarkable that PDF governs all these aspects of fly behavior given the fact that it is typically expressed in only 24 persistent adult neurons [28]: the 16 vLNs, four large abdominal neurons (l-Ab), and four small abdominal neurons (s-Ab). Though there is a growing body of evidence that the s-vLNs are centrally important for the normal control of locomotor rhythms (e.g., [29], [12], [13], [14], [15], it is by no means clear that all of PDF's circadian functions emanate only from the s-vLNs. Indeed, recent work has implicated the l-vLNs in the control of sleep and arousal and in the transduction of light-input into the clock neuron circuit [25], [26], [27] Though these studies addressed cell-type-specific functions for the two classes of PDF-positive vLN, they did not directly determine if these were due to cell-type-specific functions for PDF. Furthermore, a possible circadian role for PDF released from the l- and s-Ab PDF neurons has not been ruled-out.

To what extent are PDF's various functions distributed throughout the four classes of PDF neuron? The use of RNA-interference (RNAi) for targeted knockdown of gene expression has made it possible to address cell specific gene function in *Drosophila* [30] when used in conjunction with the Gal4/UAS system of cell-type-specific transgene expression [31]. RNAi-mediated knockdown of PDF in specific subsets of the 24 PDF expressing neurons would complement the use of Gal4/uas-driven pro-apoptotic genes or effectors of membrane excitability (e.g., [25], [26], [27]), since RNAi should, in principle, have little or no effects on neurons that do not normally express PDF. For example, the use of PDF RNAi should make experiments using widely expressed Gal4 lines easier to interpret, since off-target effects (i.e., effects on non-PDF neurons) will be less likely compared to transgenes that effect cell-death or membrane properties (see Discussion).

To begin an investigation of how PDF's established behavioral functions are distributed anatomically, we have used targeted PDF RNAi in conjunction with the Gal4/UAS system to knock-down PDF peptide expression in specific subsets of PDF neuron classes and gauged the effects on locomotor activity rhythms under LD 12∶12 and DD conditions. The specific knockdown of PDF sub-classes of PDF neuron reveals anatomically specific functions for this peptide in the control of circadian locomotion. Our results indicate that some PDF functions emanate solely from the s-vLNs while others are distributed across the vLNs. PDF released from the l- and s-Ab cells appears to have no role in the control locomotor rhythms.

## Results

### PDF RNAi within the large and small vLNs produces a phenocopy of the behaviors seen in *Pdf^01^* mutant flies

**Figure 1:**
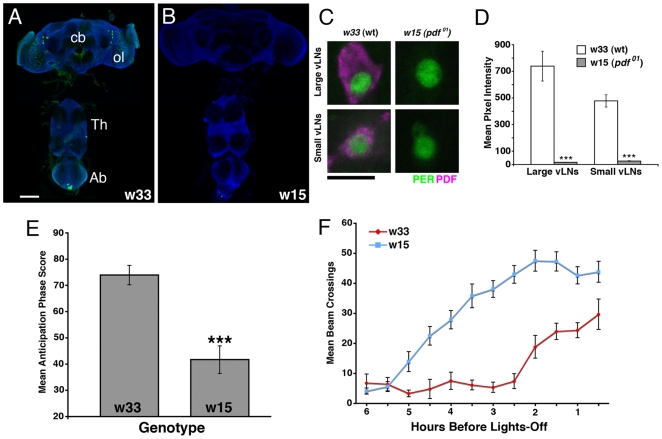
The effects of the *Pdf^01^* mutation on PDF expression and the daily pattern of locomotion under LD 12∶12. **A.** PDF expression in a wild-type (w33) CNS. PDF is shown in green, ELAV is shown in blue. cb  =  central brain, ol  =  optic lobe, Th  =  Thoracic ganglion, Ab  =  abdominal ganglion, scale bar  = 50 microns. **B.** The same PDF staining for a *Pdf^01^* (w15) mutant CNS. No PDF IR is detectable (Renn et al. 1999). **C.** Representative confocal optical sections through single l- and s-vLNs from w33 and w15 brains. PER immunostaining is shown in green, PDF in magenta. Scale bar  = 10 microns. **D.** Quantification of PDF-IR in the l- and s-vLNs from w33 and w15 brains. Values are based on optical sections of 4 vLNs from 5 brains for each genotype. A student's t-test revealed a significant difference in l- and s-vLN PDF IR between genotypes (p<0.0001). **E.** Comparison of morning anticipation phase score between w33 and w15 flies under LD 12∶12. A Student's t-test revealed a significant difference between genotypes (p<0.0001). **F.** Summary of evening activity expressed as mean beam crossings per 30 minute bin during the last six hours of the day under LD 12∶12 for w15 mutants and w33 controls. w15 flies displayed higher locomotor activity in the hours leading up to lights-off. This was reflected in a significantly advanced peak of evening activity relative to w33 controls (Table 1).

As originally described by Renn *et al.* [17], *Pdf^01^* mutants lack PDF expression (Figure 1A–D), display reduced morning anticipation and an advanced evening peak under LD 12∶12 (Figure 1E–F, Table 1, and Supplemental Figure S1), and high levels of arhythmicity and a shortened τ under DD (Table 1). We wondered if PDF's various circadian functions might be mapped to discrete subclasses of PDF-expressing neuron using targeted RNA-interference (RNAi) knockdown of PDF expression.

**Table 1:** Locomotor behavior summary for the *pdf^01^* mutant and RNAi control lines.

Genotype	n	n Rhythmic	% Rhythmic	τ (h) ± s.e.m.	Power ± s.e.m.	Evening Phase (ZT) ± SEM (min)
*;;w33 (wild-type)*	96	81	84.4	24.0±0.04	38.6±2.5	12:01±9.7
*;;w15 (pdf^01^)*	105	51	48.6	22.9±0.07	45.7±4.3	11:04±4.6
*w^1118^;;*	119	101	84.9	24.0±0.12	50.8±3.5	10:57±3.2
*y w,Dcr2;;*	71	64	90.1	23.6±0.05	65.7±4.8	10:43±3.6
*;;uas-PDFrnai/+*	31	30	96.8	23.6±0.05	97.1±7.3	11:00±3.3
*;;uas-PDFrnai*	60	49	81.7	23.7±0.07	69.9±5.9	10:38±2.8
*y w,uas-Dcr2;;uas-PDFrnai/+*	64	61	95.3	23.7±0.04	70.4±4.7	10:28±2.4
*y w,uas-Dcr2;;uas-PDFrnai*	62	55	88.7	25.8±0.25	72.0±5.5	12:04±9.3

Mutants lacking *Pdf* display several changes in locomotor rhythms as first described by Renn *et al.,* (1999). *Pdf^01^* mutants display higher levels of arhythmicity under DD, a shortened free-running period (p<0.0001 by t-test), and an advanced evening peak phase compared to wild-type flies (p<0.0001 by t-test). However, there was no significant difference between these genotypes in the power of the locomotor rhythm of rhythmic flies (p = 0.130) under the experimental conditions used here. All control lines displayed high levels of DD rhythmicity. The *uas-Dcr2* and *uas-PDFrnai* elements had little if any effects on locomotor rhythms when present as single copies. However the combination of *uas-Dcr2* and two copies of *uas-PDFrnai* resulted in an increased period and significantly delayed evening peak. One-way ANOVA of free-running period indicated a significant difference between genotypes, and a Tukey's multiple comparison test revealed a significant difference between *w^1118^* and *uas-dcr2* (p<0.05) and between *uas-Dcr2/y;;uas-PDFrnai* and all other genotypes. These control lines also displayed significant differences in the phase of evening activity (p<0.0001 by ANOVA), with Tukey's test revealing significant differences for all pairs of genotype except *w^1118^* versus *uas-Dcr2*).

**Figure 2:**
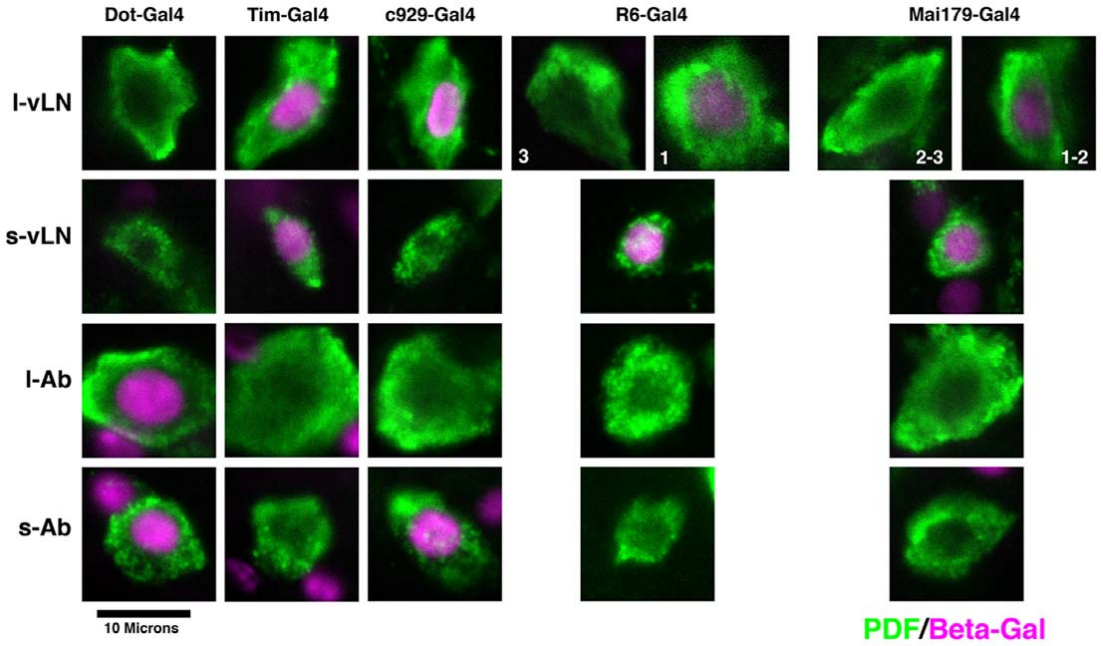
Differential expression of various Gal4 elements among the four classes of persistent adult PDF neurons. The Gal4 lines used are indicated above each column and in all cases drove *uas-lacZ^nls^* expression. Neuron type is indicated on the left. Each panel is a single confocal optical section through an individual PDF neuron; anti-PDF IR is shown in green, anti-beta-galactosidase is shown in magenta. Both *R6-Gal4* and *Mai179-Gal4* were predominately expressed in the s-vLNs, but were often weakly expressed in one or two l-vLNs, as indicated by the numbers in the top row of optical sections.

Toward this end, we obtained a series of second chromosome Gal-4 lines that are differentially expressed in the various subsets of PDF neurons. While all of these elements drove expression in non-PDF neurons, often hundreds of them (Figure S2), they also displayed useful differences in the extent of their overlap with the four classes of PDF neuron (Figure 2). Specifically, we used *tim-Gal4* to manipulate PDF levels in both classes of vLN, *Dot-Gal4* for both sets of Ab PDF neurons, and *R6-Gal4* and *Mai179-gal4* to manipulate levels primarily in the s-vLNs, but also in subsets of the l-vLNs (Figure 2). We used *c929-Gal4* to manipulate levels in the l-vLNs and s-Ab PDF neurons. This element was also expressed in the tritocerbral PDF cells of late pupae and newly emerged adults, however these neurons undergo apoptosis shortly after adult emergence and were therefore not present in the adult flies used for behavioral experiments ([28]; P.H. Taghert, unpublished data).

**Figure 3:**
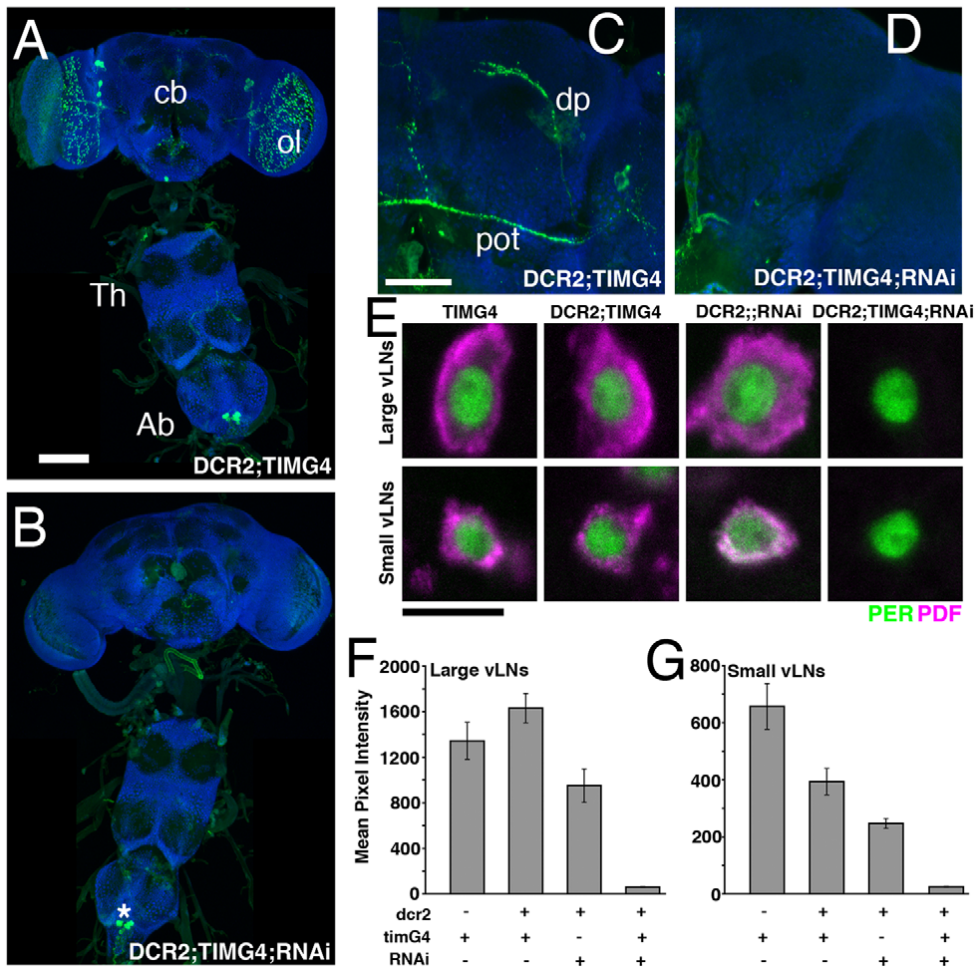
*tim-gal4-*driven *uas-Dcr2* and *uas-PDFrnai* expression knocks-down PDF specifically in the vLNs. **A**. PDF expression in a *y w,uas-Dcr2/y;tim-Gal4/+* CNS. PDF is shown in green, ELAV is shown in blue. cb  =  central brain, ol  =  optic lobe, Th  =  Thoracic ganglion, Ab  =  abdominal ganglon, scale bar  = 50 microns. **B**. PDF expression in a *y w,uas-Dcr2/y;tim-Gal4/+;uas-PDFrnai/+* CNS. These flies lacked PDF expression in the central brain, but retained abdominal PDF expression (indicated by asterisk). **C**. Confocal Z-series of PDF IR in the posterior optic tract (pot) and dorsal projection (dp) of the vLNs in a *y w,uas-Dcr2/y;tim-gal4/+*; brain. PDF is shown in green and ELAV in blue. Scale bar  = 50 microns. **D**. An equivalent Z-series for a *y w,uas-Dcr2;tim-Gal4/+;uas-PDFrnai/+* brain. Both projections lack PDF IR in these flies. **E**. Single, representative confocal optical sections through the l- and s-vLNs of *tim-Gal4/+, y w,uas-Dcr2/y;tim-Gal4/+, y w,uas-Dcr2/y;;uas-PDFrnai/+*, and *y w,uas-Dcr2/y;tim-Gal4/+;uas-PDFrnai/+* brains. PER immunostaining is shown in green, PDF in magenta. The l- and s-vLNs of *y w,uas-Dcr2/y;tim-Gal4/+;uas-PDFrnai/+* brains lacked somatic PDF IR. Scale-bar  = 10 microns. **F** and **G**. Quantification of PDF IR in the large (F) and small (G), vLNs from the genotypes shown in E. The presence or absence of the various Gal4/uas elements is indicated below the graphs. Values are based on optical sections of 4 vLNs from 5 brains for each genotype. ANOVA revealed a significant difference in somatic PDF IR for both the l- and s-vLNs among these genotypes (p<0.0001 for both cell types). A Tukey's multiple comparison test revealed that y w, uas-Dcr2;tim-Gal4/+;uas-PDFrnai/+ flies had significantly lower PDF-IR in the vLNs than all other genotypes (p<0.01) for all pair-wise comparisons for both the l- and s-vLNs. The *y w, uas-Dcr2;;uas-PDFrnai/+* flies also displayed PDF-IR that was significantly lower than the *y w, uas-Dcr2;;uas-PDFrnai* controls in both the l- and s-vLNs (p<0.01 for all pair-wise comparisons.

The l- and s-vLNs are the only PDF neurons that express the molecular clockwork and are thought to be the source of the PDF required for normal circadian behavior (Figure 2; Helfrich-Förster, personal communication). We therefore predicted that knockdown of PDF only in these classes of PDF neuron would mimic the genetic loss of *Pdf* from all cells with regard to circadian behavior. *Tim-Gal4*-driven expression of *uas-Dcr2* and *uas-PDFrnai* resulted in a clear loss of PDF-IR in the l- and s-vLNs, but spared abdominal PDF (Figure 3A–B). Confocal Z-series reconstruction of the major brain projections of the l- and s-vLNs from *uas-Drc2/y;tim-gal4/+;uas-PDFrnai/+* flies revealed a clear loss of PDF in these brain regions (Figure 3C and D). Furthermore, single optical sections through the vLN soma revealed a large and statistically significant knockdown of somatic PDF-IR in *uas-Drc2/y;tim-gal4/+;uas-PDFrnai/+* flies compared to controls (Figure 3E–G). The levels of PDF present in the soma of *uas-Dcr2/y;tim-gal4/+;uas-PDFrnai/+* vLNs were similar to those measured in *Pdf^01^* mutants using the same methods (Compare Figure 3E–G to 1C–D). Thus, PDF knockdown in the vLNs by *tim-gal4*-directed *uas-PDFrnai* and *uas-Dcr2* was complete or nearly so.

**Figure 4:**
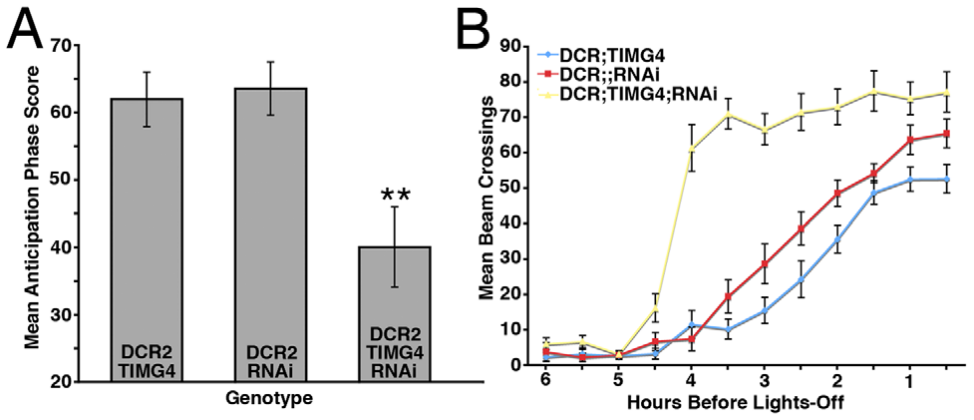
RNAi knockdown of PDF in the l- and s-vLNs recapitulates *Pdf^01^'s* LD phenotypes. **A.** Comparison of morning anticipation phase score between *uas-Dicer2;tim-gal4/+;uas-PDFrnai/+* flies and controls under LD 12∶12. ANOVA revealed a significant difference between genotypes (p<0.001) and a Tukey's multiple comparison test revealed significant differences between *uas-Dicer2;tim-gal4/+;uas-PDFrnai/+*and both controls with no significant difference between controls. **B.** Summary of evening activity expressed as mean beam crossings per 30 minute bin during the last six hours of the day under LD 12∶12 for *uas-Dicer2;tim-gal4/+;uas-PDFrnai/+* flies and controls. *uas-Dicer2;tim-gal4/+;uas-PDFrnai/+* flies displayed increased locomotor activity in the hours leading up to lights-off. This was reflected in a significantly advanced peak of evening activity relative to controls (Table 2).

In addition to a loss of PDF-IR in the vLNs, *uas-Drc2/y;tim-gal4/+;uas-PDFrnai/+*flies displayed the syndrome of circadian behavioral phenotypes originally described for *Pdf^01^* [17]. Compared to control lines, the *uas-Drc2/y;tim-gal4/+;uas-PDFrnai/+* flies showed increased arrhythmicity and a shortened τ under DD conditions (Table 2). Under LD conditions, these flies displayed reduced morning anticipation (Figures 4A and S3) and an increase in activity levels in the six hours before lights-off (Figure 4B), which resulted in an advanced evening peak (Table 2 and Figure S3). Thus, loss of PDF only from the l- and s-vLNs was sufficient to provoke the behavioral phenotypes associated with the *Pdf^01^* mutation, despite the presence of PDF in the abdominal PDF cells.

**Table 2:** Locomotor behavior summary for *tim-Gal4* mediated RNAi knockdown of PDF.

Genotype	n	n Rhythmic	% Rhythmic	τ (h) ± s.e.m.	Power ± s.e.m.	Evening Phase (ZT) ± SEM (min)
*;tim-Gal4/+;*	21	19	90.48	24.1±0.10	49.5±6.9	10:51±4.0
*y w,uas-Dcr2;tim-Gal4/+*	29	29	100.00	24.2±0.06	88.0±5.6	11:05±5.1
*y w,uas-Dcr2;;uas-PDFrnai/+*	64	61	95.3	23.7±0.04	70.4±4.7	10:28±2.4
*y w,uas-Dcr2;tim-Gal4/+;uas-PDFrnai/+*	80	41	51.25	22.6±0.10	37.1±3.3	10:10±2.9

Loss of PDF from the l- and s-vLNs by *tim-Gal4*-driven expression of *uas-Dcr2* and *uas-PDFrnai* was associated with the major locomotor rhythm effects of *Pdf^01^* under DD and LD 12∶12 conditions. *y w*,*uas-Dcr2/y;tim-Gal4/+;uas-PDFrnai/+* flies displayed reduced rhythmicity under DD relative to all three control lines. ANOVA revealed a significant difference in τ between these four genotypes (p<0.0001) and a Tukey's test revealed that *y w*,*uas-Dcr2/y;tim-Gal4/+;uas-PDFrnai/+* flies had a significantly shorter free-running period than all other genotypes (p<0.001 in for all comparisons). ANOVA also revealed a significant difference between genotypes in evening peak phase in LD 12∶12 (p<0.0001) with *y w*,*uas-Dcr2/y;tim-Gal4/+;uas-PDFrnai/+* flies displaying a significantly early peak relative to all other genotypes (Tukey's test, p<0.001 for all comparisons).

### PDF is not required in the abdominal PDF neurons for normal locomotor rhythms under LD and DD conditions

**Figure 5:**
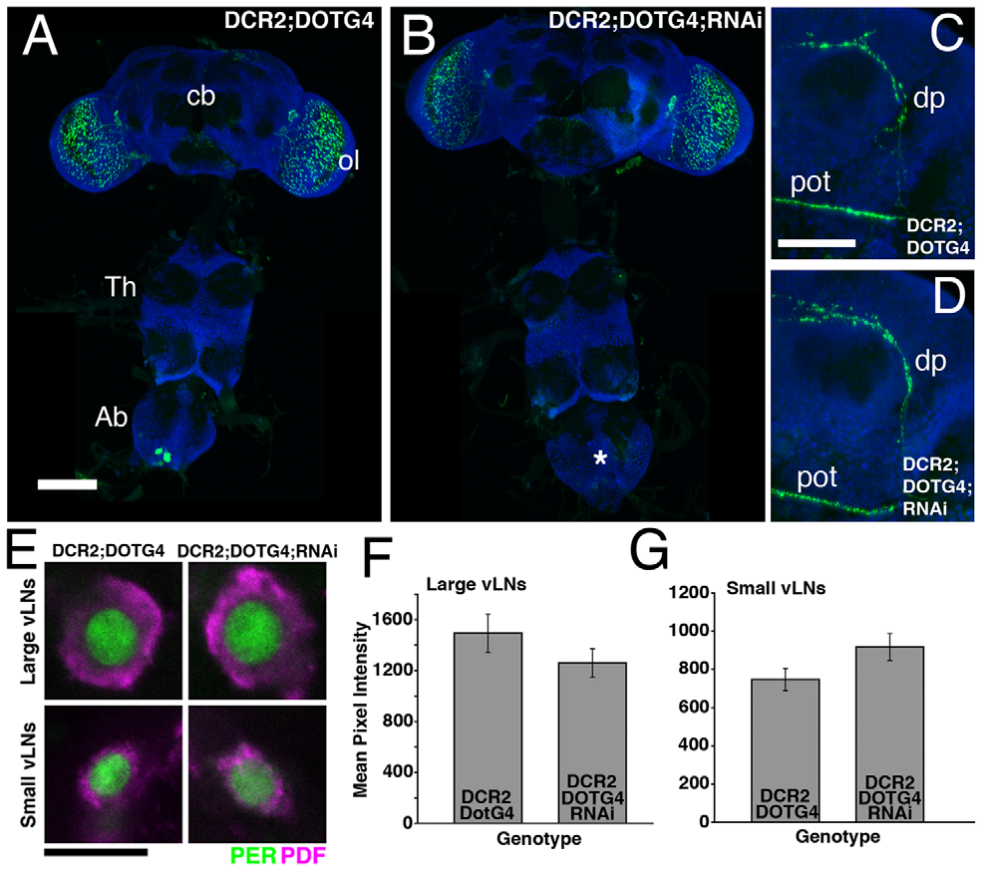
*dot-Gal4-*driven *uas-Dcr2* and *uas-PDFrnai* expression knocks-down abdominal PDF but does not effect vLN PDF levels. **A**. PDF expression in a *y w,uas-Dcr2/y;dot-Gal4/+* CNS. PDF is shown in green, ELAV is shown in blue. cb  =  central brain, ol  =  optic lobe, Th  =  Thoracic ganglion, Ab  =  abdominal ganglion, scale bar  = 50 microns. **B**. PDF expression in a *y w,uas-Dcr2/y;dot-Gal4/+;uas-PDFrnai/+* CNS. These lacked PDF expression in the abdominal ganglion as indicated by the asterisk. This is shown at higher resolution in Supplemental Figure S4. These flies retained PDF expression in the brain. **C**. Confocal Z-series of PDF IR in the posterior optic tract (pot) and dorsal projection (dp) of the vLNs in a *y w,uas-Dcr2/y;dot-gal4/+*; brain. PDF is shown in green and ELAV in blue. Scale bar  = 50 microns. **D**. An equivalent Z-series for a *y w,uas-Dcr2;dot-Gal4/+;uas-PDFrnai/+* brain showing normal PDF expression in these projections. **E**. Single, representative confocal optical sections through a l- and s-vLN from *y w,uas-Dcr2/y;dot-Gal4/+* and *y w,uas-Dcr2/y;dot-Gal4/+;uas-PDFrnai/+* brains. PER immunostaining is shown in green, PDF in magenta. Both genotypes showed clear somatic PDF IR in both vLN subtypes. Scale-bar  = 10 microns. **F** and **G**. Quantification of PDF IR in the large (F) and small (G), vLNs from the genotypes shown in E. The presence or absence of the various Gal4/uas elements is indicated on the graphs. Values are based on optical sections of 4 vLNs from 5 brains for each genotype. ANOVA revealed no significant difference in somatic PDF IR for either the l- and s-vLNs between these genotypes (p = 0.206 for the l-vLNs; p = 0.063 for s-vLNs).

Though PDF's effects on circadian rhythms appear to emanate from the vLNs, no experiment has yet determined if PDF released from the abdominal PDF neurons contributes to normal locomotor rhythms. We therefore asked if RNAi-mediated PDF knockdown in the abdominal PDF neurons would have measurable effects on locomotor behavior. *Dot-Gal4* driven *uas-Dcr2* and *uas-PDFrnai* expression resulted in a large reduction of abdominal PDF-IR (Figures 5A–B and S4), with no measurable effects on PDF-IR in the projections or soma of the l- and s-vLNs (Figure 5C–G).

**Figure 6:**
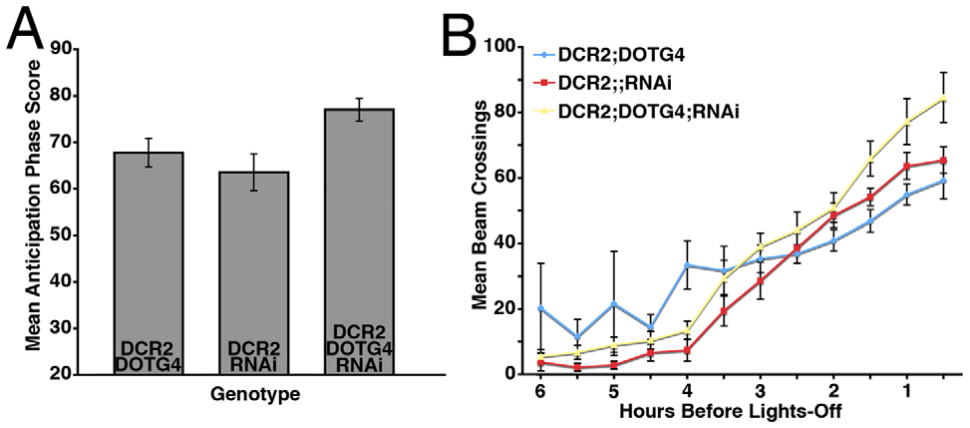
RNAi knockdown of PDF in the l- and s-Ab cells has no *Pdf^01^*-like effects on locomotor behavior under LD. **A.** Comparison of morning anticipation phase score between *uas-Dicer2;Dot-gal4/+;uas-PDFrnai/+* flies and controls under LD 12∶12. ANOVA revealed significant differences between genotypes (p = 0.0107) with *uas-Dicer2;Dot-gal4/+;uas-PDFrnai/+* flies displaying a higher score compared to controls, but a Tukey's multiple comparison test revealed no significant difference between *uas-Dicer2;Dot-gal4/+;uas-PDFrnai/+*and the *uas-Dicer2;Dot-Gal4/+* control. **B.** Summary of evening activity expressed as mean beam crossings per 30 minute bin during the last six hours of the day under LD 12∶12 for *uas-Dicer2;Dot-gal4/+;uas-PDFrnai/+* flies and controls. *uas-Dicer2;Dot-gal4/+;uas-PDFrnai/+* flies displayed no *Pdf^01^*-like increases in locomotor activity in the hours leading up to lights-off and there was no correlation between evening peak phase and PDF levels within the Ab PDF neurons (Table 3).

Despite this large reduction in abdominal PDF levels, *y w,uas-Dcr2/y;dot-Gal4/+;uas-PDFrnai/+* flies were robustly rhythmic with a normal τ under DD (Table 3). These flies also displayed clear morning anticipation, a normally phased evening peak under LD conditions (Figure 6, Table 3, and Figure S3). Thus, locomotor rhythms did not depend on normal PDF expression in the l- and s-Ab neurons.

**Table 3:** Locomotor behavior summary for *dot-Gal4* mediated RNAi knockdown of PDF.

Genotype	n	n Rhythmic	% Rhythmic	τ (h) ± s.e.m.	Power ± s.e.m.	Evening Phase (ZT) ± SEM (min)
*;Dot-Gal4/+;*	29	25	86.2	23.7±0.07	59.0±3.9	11:22±4.8
*y w,uas-Dcr2;Dot-Gal4/+;*	44	43	97.7	23.5±0.04	94.9±4.5	11:14±4.8
*y w, uas-Dcr2;;uas-PDFrnai/+*	64	61	95.3	23.7±0.04	70.4±4.7	10:28±2.4
*y w, uas-Dcr2;Dot-Gal4/+;uas-PDFrnai/+*	55	51	92.7	23.5±0.04	104.3±5.4	10:51±4.7

Loss of abdominal PDF expression by *Dot-Gal4*-driven *uas-PDFrnai* and *uas-Dcr2* did not result in a decrease in rhythmicity under DD. ANOVA revealed a significant difference between genotypes in τ, but a Tukey's test did not detect differences between *y w,uas-Dcr2;Dot-Gal4/+;uas-PDFrnai/+* flies and controls (p>0.05 for each pair-wise comparison). ANOVA revealed a difference between genotypes in the power of free-running rhythms (ANOVA p<0.0001) and in evening peak phase under LD (ANOVA p<0.0001), however these differences reflected the differences observed between *y w,uas-Dcr2;Dot-Gal4/+* and *y w,uas-Dcr2/y;;uas-PDFrnai/+* and were not correlated with PDF levels in the abdominal PDF neurons.

### Knockdown of PDF in the large vLNs has no significant effects on free-running or entrained locomotor rhythms

The l-vLNs do not maintain a free-running rhythm of clock protein expression or sub-cellular localization under DD conditions [32], [33] and are not thought to play a role in the maintenance of free-running locomotor rhythms. Nevertheless, a potential role of PDF released from the l-vLNs in the maintenance of free-running rhythms has not been ruled-out. Furthermore, recent work has suggested a role for the l-vLNs in the regulation of daytime sleep and arousal [25], [26], [27] and for light input to the circadian network [27], [34]. We therefore asked if knockdown of PDF in the l-vLNs would have measurable effects on locomotor rhythms under DD and LD conditions.

**Figure 7:**
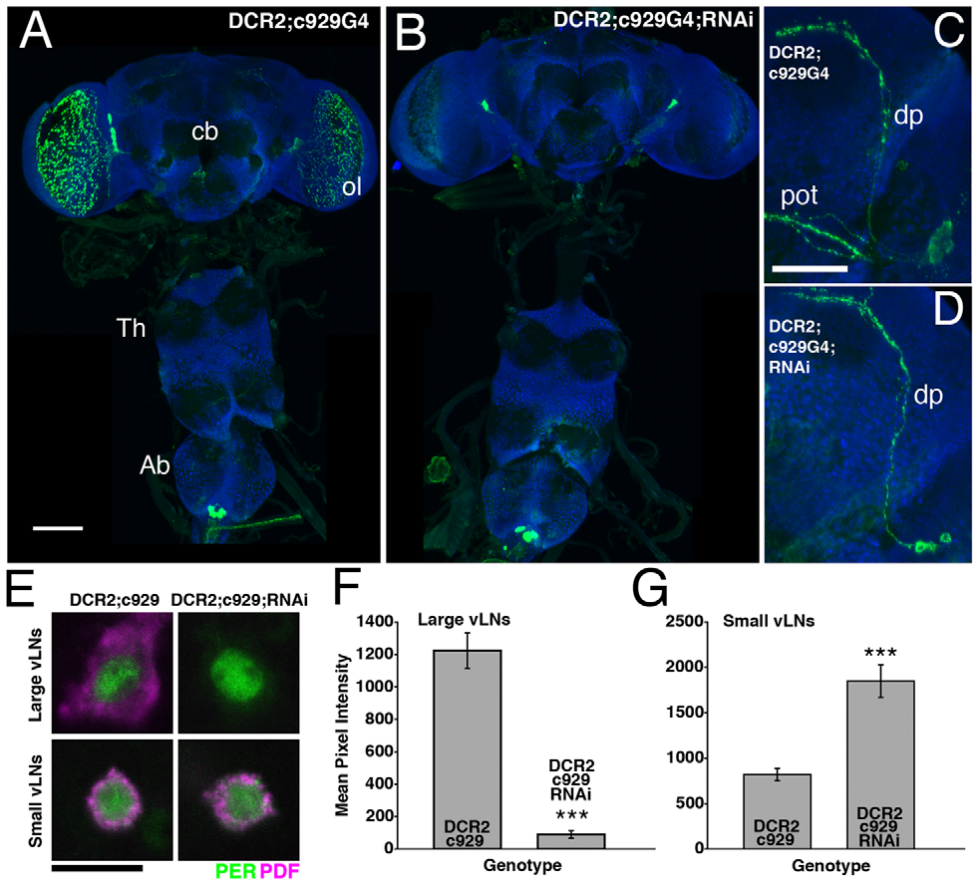
*c929-gal4*-driven *uas-Dcr2* and *uas-PDFrnai* expression results in a knockdown of l-vLN PDF and increased PDF IR in the s-vLNs. **A**. PDF expression in a *y w,uas-Dcr2/y;c929-Gal4/+* CNS. PDF is shown in green, ELAV is shown in blue. cb  =  central brain, ol  =  optic lobe, Th  =  Thoracic ganglion, Ab  =  abdominal ganglion, scale bar  = 50 microns. **B**. PDF expression in a *y w,uas-Dcr2/y;c929-Gal4/+;uas-PDFrnai/+* CNS. These lacked PDF expression in the l-vLN soma and optic lobe projections, but retained s-vLN and l-Ab PDF expression. Most CNSs of this genotype lacked PDF IR in the s-Ab neurons (Supplemental Figure S4). **C**. Confocal Z-series of PDF IR in the posterior optic tract (pot) and dorsal projection (dp) of the vLNs in a *y w,uas-Dcr2/y;c929-gal4/+* brain. PDF is shown in green and ELAV in blue. Scale bar  = 50 microns. **D**. An equivalent Z-series for a *y w,uas-Dcr2;c929-Gal4/+;uas-PDFrnai/+* brain. PDF IR is not detectable in the pot of these flies but is retained in the dp. **E**. Single, representative confocal optical sections through a l- and s-vLN from *y w,uas-Dcr2/y;c929-Gal4/+* and *y w,uas-Dcr2/y;c929-Gal4/+;uas-PDFrnai/+* brains. PER immunostaining is shown in green, PDF in magenta. The l-vLNs of the latter genotype lacked PDF IR. PDF IR was apparent in the s-vLNs of both genotypes. Scale-bar  = 10microns. **F** and **G**. Quantification of PDF IR in the large (F) and small (G), vLNs from the genotypes shown in E. The presence or absence of the various Gal4/uas elements is indicated on the graphs. Values are based on optical sections of 4 vLNs from 5 brains for each genotype. A Student's T-test revealed significantly lower PDF IR in the l- vLNs of *y w,uas-Dcr2/y;c929-Gal4/+;uas-PDFrnai/+* brains (p<0.0001). This genotype also showed significantly higher PDF IR in the s-vLNs (p<0.0001).

**Figure 8:**
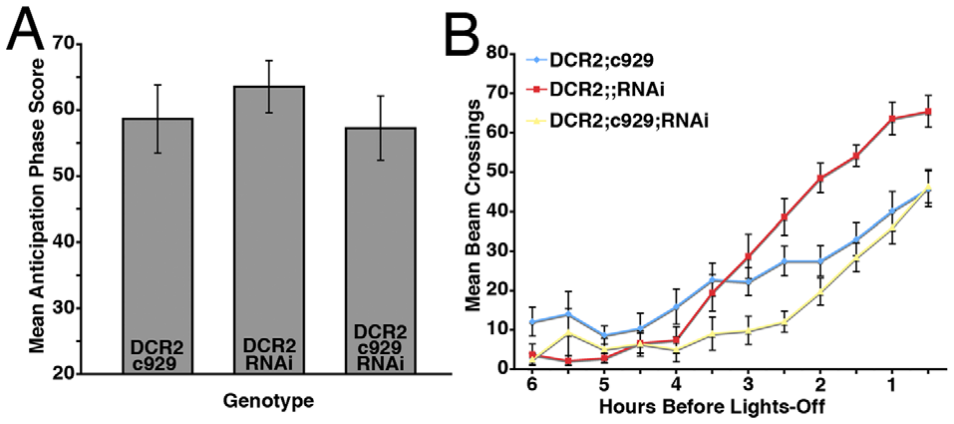
RNAi knockdown of PDF in the l-vLNs has no *Pdf^01^*-like effects on locomotor behavior under LD. **A.** Comparison of morning anticipation phase score between *uas-Dicer2;c929-gal4/+;uas-PDFrnai/+* flies and controls under LD 12∶12. ANOVA revealed no significant differences between genotypes (p = 0.58). **B.** Summary of evening activity expressed as mean beam crossings per 30 minute bin during the last six hours of the day under LD 12∶12 for *uas-Dicer2;c929-gal4/+;uas-PDFrnai/+* flies and controls. *uas-Dicer2;c929-gal4/+;uas-PDFrnai/+* flies displayed no *Pdf^01^*-like increases in locomotor activity in the hours leading up to lights-off and here was no significant effect of PDF loss from the l-vLNs on the evening peak phase (Table 4).

PDF knockdown using *c929-Gal4* resulted in a clear loss of PDF IR from both the l-vLNs (Figure 7A–F) and the s-Ab neurons (Supplemental Figure S4). *c929-gal4*-driven *uas-Dcr2* and *uas-PDFrnai* expression resulted in a loss of PDF from both major l-vLN projections (the optic lobes and pot; Figure 7A–D) and l-vLN soma (Figure 7E–F). Quantification of PDF-IR in the l-vLNs revealed a near complete loss of peptide (Figure 7F). The loss of PDF in the l-vLNs was accompanied by a significant increase in PDF levels in the s-vLNs (Figure 7E and G). Despite these changes in vLN PDF IR, *y w,uas-Dcr2/y;c929-Gal4/+;uas-PDFrnai/+* flies showed normal locomotor rhythms under both DD and LD conditions (Table 4 and Figures 8 and S3). Thus, normal PDF expression was not required in the l-vLNs for normal locomotor rhythms and under DD and LD conditions.

**Table 4:** Locomotor behavior summary for *c929-Gal4-*mediated RNAi knockdown of PDF.

Genotype	n	n Rhythmic	% Rhythmic	τ (h) ± s.e.m.	Power ± s.e.m.	Evening Phase (ZT) ± SEM (min)
;c929-Gal4/+;	63	57	90.5	23.8±0.06	44.8±2.9	10:57±3.8
*y w, uas-*Dcr2;c929-Gal4/+;	47	34	72.3	24.1±0.10	70.4±4.7	10:28±2.4
*y w, uas-*Dcr2;;uas-PDFrnai/+	64	61	95.3	23.7±0.04	70.4±4.7	10:28±2.4
*y w, uas-*dcr2;c929-Gal4/+;uas-PDFrnai/+	44	34	79.2	23.5±0.06	46.1±3.4	10:41±6.8

*c929-Gal4*-driven expression of *uas-Dcr2* appeared to reduce DD rhythmicity (see values for *y w,uas-Dcr2/y;;* in Table 1), although the majority of these flies maintained free-running locomotor rhythms. Loss of PDF from the l-vLNs and s-Ab PDF neurons in *y w,uas-Dcr2/y;c929-Gal4/+;uas-PDFrnai/+*flies was not associated with a significant change of τ relative to controls. Although ANOVA revealed significant differences in τ between genotypes (p = 0.0023), a Tukey's test revealed no significant difference between *y w,uas-Dcr2/y;c929-Gal4/+;uas-PDFrnai/+* flies and *y w,uas-Dcr2/y;;uas-PDFrnai/+* controls. ANOVA also revealed significant differences in power between genotypes, but a Tukey's test revealed no significant difference between *y w,uas-Dcr2/y;c929-Gal4/+;uas-PDFrnai/+* flies and *y w,uas-Dcr2/y;;uas-PDFrnai/+* controls. Similarly, ANOVA revealed significant differences in evening peak phase between genotypes (p<0.0001), but a Tukey's test revealed that *y w,uas-Dcr2/y;c929-Gal4/+;uas-PDFrnai/+*flies did not differ significantly from controls (p>0.05 for each pair-wise comparison).

### Knockdown of PDF in the small vLNs results in DD arhythmicity and a reduction in morning anticipation under LD

**Figure 9:**
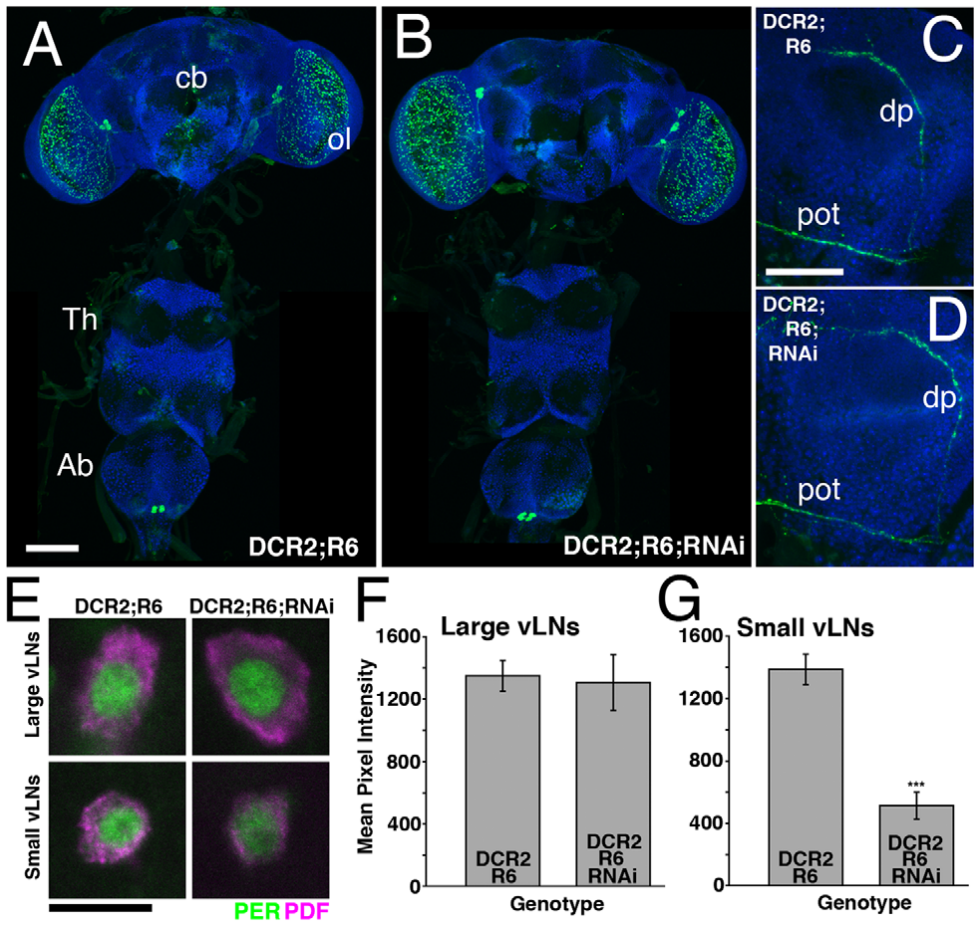
*R6-Gal4*-driven *uas-Dcr2* and *uas-PDFrnai* expression results in partial PDF-knockdown specifically in the s- vLNs and does not effect PDF levels in the l-vLNs. **A**. PDF expression in a *y w,uas-Dcr2/y;R6-Gal4/+* CNS. PDF is shown in green, ELAV is shown in blue. cb  =  central brain, ol  =  optic lobe, Th  =  Thoracic ganglion, Ab  =  abdominal ganglion, scale bar  = 50 microns. **B**. PDF expression in a *y w,uas-Dcr2/y;R6-Gal4/+;uas-PDFrnai/+* CNS. At this magnification PDF expression looks normal for all PDF neuron classes. **C**. Confocal Z-series of PDF IR in the posterior optic tract (pot) and dorsal projection (dp) of the vLNs in a *y w,uas-Dcr2/y;R6-gal4/+* brain. PDF is shown in green and ELAV in blue. Scale bar  = 50 microns. **D**. An equivalent Z-series for a *y w,uas-Dcr2;R6-Gal4/+;uas-PDFrnai/+* brain. PDF IR is present in both the dp and pot of these flies. **E**. Single, representative confocal optical sections through the l- and s-vLNs of *y w,uas-Dcr2/y;R6-Gal4/+* and *y w,uas-Dcr2/y;R6-Gal4/+;uas-PDFrnai/+* brains. PER immunostaining is shown in green, PDF in magenta. The s-vLNs of the latter genotype had relatively low somatic PDF IR. **F** and **G**. Quantification of PDF IR in the large (F) and small (G) vLNs from the genotypes shown in E. The presence or absence of the various Gal4/uas elements is indicated on the graphs. Values are based on optical sections of 4 vLNs from 5 brains for each genotype. Student's T-test revealed a significantly lower PDF IR in the s-vLNs of *y w,uas-Dcr2/y;R6-Gal4/+;uas-PDFrnai/+* brains (p<0.0001), but no significant difference in l-vLN PDF IR (p = 0.8365).

A large body of evidence indicates that the s-vLNs are critical neuronal pacemakers for the maintenance of free-running locomotor rhythms in *Drosophila* (reviewed in [7]). We therefore wondered how these rhythms would be affected by knockdown of PDF in the s-vLNs. *R6-Gal4* is expressed in the s-vLNs and in relatively few other neurons in the CNS ([35] Supplemental Figure S2). This non-s-vLN expression typically includes relatively weak expression in a single l-vLN per hemisphere ([35]; Figure 2). *R6-Gal4*-driven *uas-Dcr2* and *uas-PDFrnai* did not result in any obvious loss of PDF IR in any class of PDF neuron or PDF projection (Figure 9A–D). However, quantification of somatic PDF IR in the vLN soma revealed a significant (63%) reduction in PDF IR in the s-vLN of *y w,uas-Dcr2/y;R6-Gal4/+;uas-PDFrnai/+* flies with no measurable reduction in l-vLN PDF IR (Figure 9E–G).

**Figure 10:**
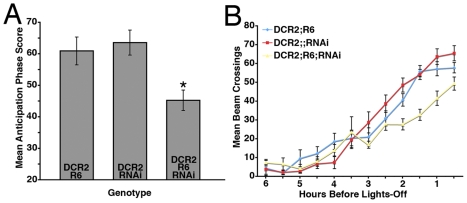
Partial RNAi knockdown of PDF in the s-vLNs results in a reduction in morning anticipation, but normal evening locomotor behavior. **A**. Comparison of morning anticipation phase score between *uas-Dicer2;R6-gal4/+;uas-PDFrnai/+* flies and controls under LD 12∶12. ANOVA revealed significant differences between genotypes (p = 0.002) and a Tukey's multiple comparison test revealed significant differences between *uas-Dicer2;R6-gal4/+;uas-PDFrnai/+* flies and both controls (p<0.05 for both pairwise comparisons) with no significant difference between the two control lines (p>0.05). **B**. Summary of evening activity expressed as mean beam crossings per 30 minute bin during the last six hours of the day under LD 12∶12 for *uas-Dicer2;R6-gal4/+;uas-PDFrnai/+* flies and controls. *uas-Dicer2;R6-gal4/+;uas-PDFrnai/+* flies did not display a *Pdf^01^*-like increase in locomotor activity in the hours leading up to lights-off relative to controls. R6-mediated reduction of PDF levels in the s-vLNs was associated with a *later* evening peak phase relative to *uas-Dicer2;R6-gal4/+;* and *uas-Dicer2;;uas-PDFrnai/+* controls, but was not significantly different from; R6-Gal4/+; controls (Table 5).

The s-vLN-specific reduction in PDF IR by *R6-Gal4*-mediated PDF RNAi was associated with high levels of arhythmicity under DD (Table 5). Nevertheless, rhythmic *y w,uas-Dcr2/y;R6-Gal4/+;uas-PDFrnai/+* flies showed a normal τ (Table 5). Under LD 12∶12, *y w,uas-Dcr2/y;R6-Gal4/+;uas-PDFrnai/+*flies displayed significantly reduced morning anticipation relative to *y w,uas-Dcr2;R6-Gal4/+* and *y w,uas-Dcr2;;uas-PDFrnai/+* controls (Figures 10A and S3) and a modestly delayed evening peak (Figure 10B and Table 5).

**Table 5:** Locomotor behavior summary for *R6-Gal4* mediated RNAi knockdown of PDF.

Genotype	n	n Rhythmic	% Rhythmic	τ (h) ± s.e.m.	Power ± s.e.m.	Evening Phase (ZT) ± SEM (min)
;R6-Gal4/+;	60	35	58.3	23.5±0.26	25.0±2.5	10:53±4.5
y w, uas-Dcr2;R6-Gal4/+;	57	53	93	23.6±0.06	53.1±5.1	10:46±4.4
y w, uas-Dcr2;;uas-PDFrnai/+	64	61	95.3	23.7±0.04	70.4±4.7	10:28±2.4
y w, uas-Dcr2;R6-Gal4/+;uas-PDFrnai/+	83	9	10.8	23.8±0.45	29.6±9.5	11:11±6.5

The *R6-Gal4* line showed relatively low levels of rhythmicity under DD conditions, but *y w,uas-Dcr2;R6-Gal4/+* flies were robustly rhythmic. *y w,uas-Dcr2/y;R6-Gal4/+;uas-PDFrnai/+* flies displayed a very high incidence of arhythmicity under DD conditions relative to control lines. ANOVA revealed significant difference in τ under DD (p<0.0001), but a Tukey's test revealed no significant difference in period between *y w,uas-Dcr2/y;R6-Gal4/+;uas-PDFrnai/+* flies and *y w,uas-Dcr2;R6-Gal4/+*and *y w,uas-Dcr2;;uas-PDFrnai/+* controls (p>0.05 for both comparisons). ANOVA revealed a significant difference in the power of free-running locomotor rhythms between genotypes, but a Tukey's test revealed no significant difference between between *y w,uas-Dcr2/y;R6-Gal4/+;uas-PDFrnai/+* flies and *y w,uas-Dcr2;R6-Gal4/+* controls (p>0.05). ANOVA revealed a significant difference in evening peak phase between genotypes and a Tukey's test revealed significant differences in phase between *y w,uas-Dcr2/y;R6-Gal4/+;uas-PDFrnai/+* flies and *y w,uas-Dcr2;R6-Gal4/+*(p<0.01) and *y w,uas-Dcr2;;uas-PDFrnai/+* controls (p<0.001). Tukey's test revealed no difference in evening peak phase between *y w,uas-Dcr2/y;R6-Gal4/+;uas-PDFrnai/+* flies and *R6-Gal4* controls.

**Figure 11:**
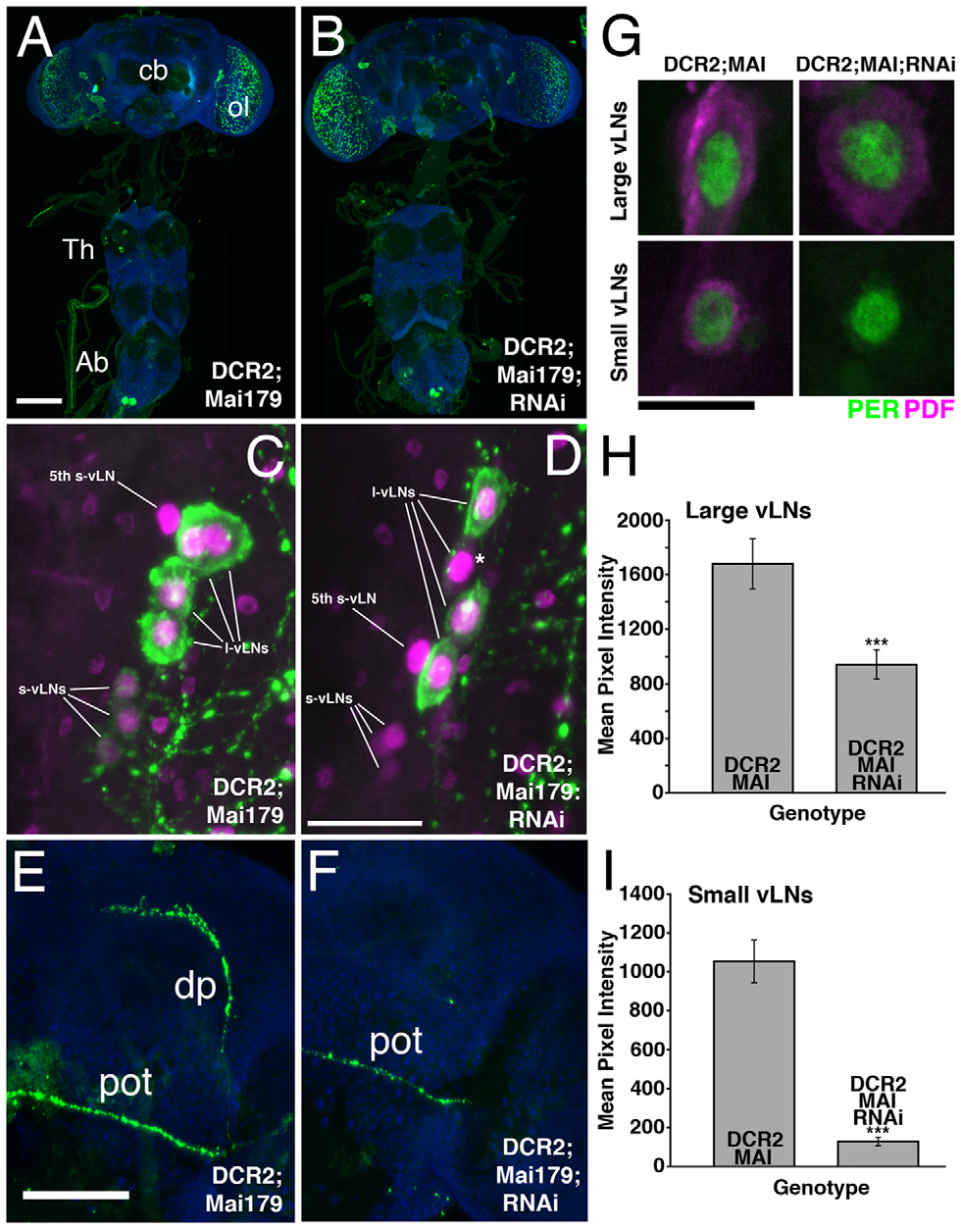
*Mai179-Gal4*-driven *uas-Dcr2* and *uas-PDFrnai* knocks down PDF in the s- vLNs and a subset of the l-vLNs. **A**. PDF expression in a *y w,uas-Dcr2/y;Mai179-Gal4/+* CNS. PDF is shown in green, ELAV is shown in blue. cb  =  central brain, ol  =  optic lobe, Th  =  Thoracic ganglion, Ab  =  abdominal ganglion, scale bar  = 50 microns. **B.** PDF expression in a *y w,uas-Dcr2/y;Mai179-Gal4/+;uas-PDFrnai/+* CNS. Only l-vLN and abdominal PDF expression was apparent in this genotype. **C.** Confocal Z-series through the vLNs of a *y w,uas-Dcr2/y;Mai179-Gal4/+*brain double labled for PDF (green) and PER (magenta). PDF IR is detectable in four l- and four s-vLNs. The PDF-negative 5^th^ s-vLN is also labeled. **D.** An equivalent Z-series through the vLNs of a *Dcr2/y;Mai179-Gal4/+;uas-PDFrnai/+* brain. These brains showed no somatic PDF IR in the s-vLNs and often contained a single l-vLN with extremely low somatic PDF IR. One such l-vLN is indicated by an asterisk. Scale Bar  = 20 microns. **E.** Confocal Z-series of PDF IR in the posterior optic tract (pot) and dorsal projection (dp) of the vLNs in a *y w,uas-Dcr2/y;Mai179-gal4/+* brain. PDF is shown in green and ELAV in blue. Scale bar  = 50 microns. **F.** An equivalent Z-series for a *y w,uas-Dcr2;Mai179-Gal4/+;uas-PDFrnai/+* brain. PDF IR is present in the pot but not the dp of these flies. **G**. Single, representative confocal optical sections through a l- and s-vLN from *y w,uas-Dcr2/y;Mai179-Gal4/+* and *y w,uas-Dcr2/y;Mai179-Gal4/+;uas-PDFrnai/+* brains. PER immunostaining is shown in green, PDF in magenta. The s-vLNs of the latter genotype displayed no obvious somatic PDF IR. Scale-bar  = 10 microns. **H** and **I**. Quantification of PDF IR in the large (H) and small (I), vLNs from the genotypes shown in G. The presence or absence of the various Gal4/uas elements is indicated on the graphs. Values are based on optical sections of 4 vLNs from 5 brains for each genotype. A Student's T-test revealed significantly lower PDF IR in the l-vLNs (p = 0.001) and s-vLNs (p<0.0001) of *y w,uas-Dcr2/y;Mai-Gal4/+;uas-PDFrnai/+* brains.

Though *R6-Gal4*-mediated reduction of PDF had measurable effects on DD rhythmicity and morning anticipation under LD, it had no clear *Pdf^01^*-like effects on τ, and evening peak phase under LD. We wondered if a lack of effects on τ and evening peak phase were a due to the remaining low levels of PDF in the s-vLNs of *y w,uas-Dcr2/y;R6-Gal4/+;uas-PDFrnai/+* flies (Figure 9E and G). We therefore sought to reduce PDF to a greater extent in the s-vLNs using another Gal4 element. *Mai179-Gal4* is expressed in all s-vLNs and in one or two l-vLNs per hemisphere ([13]; Figure 2). *Mai179-Gal4-*driven *uas-Dcr2* and *uas-PDFrnai* expression resulted in a clear reduction of PDF IR in the s-vLN soma and a loss of PDF IR in the dorsal projections of the s-vLNs (Figure 11). In most brains a single l-vLN also lacked clear PDF IR (Figure 11C–D), but the major projections of the l-vLNs looked normal (Figure 11A–B and E–F), indicating that most l-vLNs maintained PDF-positive projections in the posterior optic tract and optic lobes. Quantification of somatic PDF IR in the vLN soma revealed a clear reduction in PDF IR in both the l- and s-vLNs of *y w,uas-Dcr2/y;Mai179-Gal4/+;uas-PDFrnai/+* flies, with the s-vLNs showing a much greater reduction in PDF-IR (44% reduction for the l-vLNs and 88% reduction for the s-vLNs; Figure 11G–I). The *Mai179-Gal4*-mediated PDF knockdown in the s-vLN resulted in undetectable PDF IR in the dorsal projections of *y w,uas-Dcr2/y;Mai179-Gal4/+;uas-PDFrnai/+* flies (Figure 11F). Thus *Mai179-Gal4*-mediated knockdown of PDF in the s-vLNs was greater than that for *R6-Gal4* (Compare Figures 9 and 11), but unlike *R6-Gal4*, *Mai-179-Gal4*-mediated PDF knockdown also resulted in a measurable loss of PDF IR from the l-vLNs.

**Figure 12:**
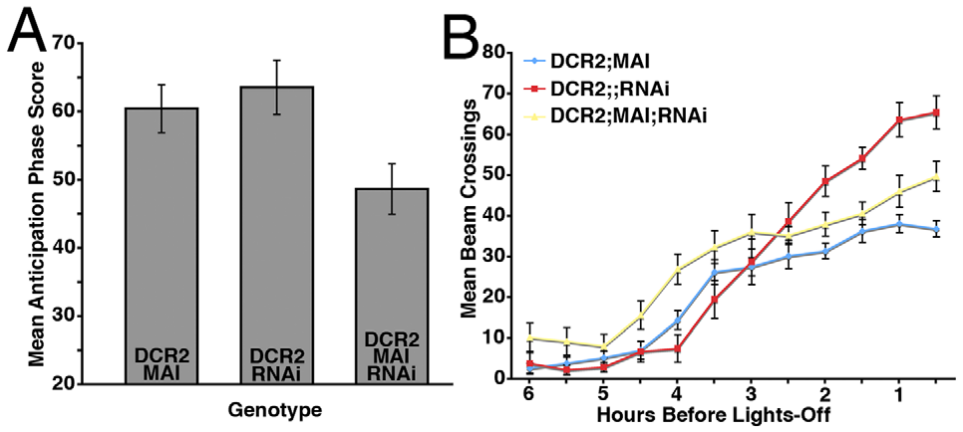
*Mai179-Gal4*-mediated PDF knockdown in the s-vLNs and in a subset of the l-vLNs has only modest effects on locomotion under LD conditions. **A.** Comparison of morning anticipation phase score between *uas-Dicer2;Mai179-gal4/+;uas-PDFrnai/+* flies and controls under LD 12∶12. ANOVA revealed significant differences between genotypes (p = 0.0167), but a Tukey's multiple comparison test revealed significant differences between *uas-Dicer2;Mai179-gal4/+;uas-PDFrnai/+* and *uas-Dicer2;;uas-PDFrnai* flies (p<0.05), but not between *uas-Dicer2; Mai179-gal4/+;uas-PDFrnai/+* and *uas-Dicer2; Mai179-gal4/+* (p>0.05). **B.** Summary of evening activity expressed as mean beam crossings per 30 minute bin during the last six hours of the day under LD 12∶12 for *uas-Dicer2;Mai179-gal4/+;uas-PDFrnai/+* flies and controls. *uas-Dicer2; Mai179-gal4/+;uas-PDFrnai/+* flies did not display a *Pdf^01^*-like increase in locomotor activity in the hours leading up to lights-off relative to controls and displayed a normal phase of evening peak activity relative to controls (Table 6).

The near complete loss of s-vLN PDF-IR and partial loss of l-vLN PDF-IR found in *y w,uas-Dcr2/y;Mai179-Gal4/+;uas-PDFrnai/+* flies was associated with high arhythmicity in DD (Table 6). Despite the significant PDF loss in the s-vLN soma and projections and a partial loss of PDF IR from the l-vLNs, rhythmic flies of this genotype displayed a normal τ under DD and a normally phased evening peak under LD (Table 6 and Figures 12B and S3). Thus, as was the case for *R6-Gal4* mediated partial PDF knockdown, the strong reduction of PDF levels in the s-vLNs had clear effects on the ability to maintain free-running locomotor rhythms, but had little or no effect on τ or on the phase of the evening peak under LD. Interestingly, *y w,uas-Dcr2/y;Mai179-Gal4/+;uas-PDFrnai/+* flies displayed reduced morning anticipation, but this effect was not statistically significant when compared to *y w,uas-Dcr2/y;;uas-PDFrnai/+* controls (Figures 12A and S3). Thus, despite the significant reduction of PDF IR in the l-vLNs, *Mai179-Gal4*–mediated knockdown of PDF did not result in a more severe morning peak phenotype (Compare Figures 10A and 12A).

**Table 6:** Locomotor behavior summary for *Mai179-Gal4* mediated RNAi knockdown of PDF.

Genotype	n	n Rhythmic	% Rhythmic	τ (h) ± s.e.m.	Power ± s.e.m.	Evening Phase (ZT) ± SEM (min)
*;Mai179-Gal4/+;*	25	21	84	23.7±0.09	38.1±4.9	10:28±8.5
*y w, uas-Dcr2;Mai179-Gal4/+;*	62	58	93.5	23.6±0.05	49.6±3.7	10:23±3.5
*y w, uas-Dcr2;;uas-PDFrnai/+*	64	61	95.3	23.7±0.04	70.4±4.7	10:28±2.4
*y w, uas-dcr2;Mai179-Gal4/+;uas-PDFrnai/+*	43	12	27.9	23.5±0.50	25.3±4.3	10:40±6.7

*Mai179-Gal4*-mediated *uas-Dcr2* and *uas-PDFrnai* expression was associated with high levels of arhythmicity under DD relative to controls. The τ of rhythmic *y w,uas-Dcr2/y;Mai179-Gal4/+;uas-PDFrnai/+* flies was not significantly different than those of *y w,uas-Dcr2/y;Mai179-Gal4/+* and *y w,uas-Dcr2/y;;uas-PDFrnai/+* controls (ANOVA p = 0.6314). ANOVA revealed a significant difference in the power of free-running locomotor rhythms between genotypes, but Tukey's test revealed no significant difference between *y w,uas-Dcr2/y;Mai179-Gal4/+;uas-PDFrnai/+* flies and *y w,uas-Dcr2/y;Mai179-Gal4/+* controls (p>0.05). There was no significant difference in evening peak phase between genotypes (ANOVA p = 0.0628).

## Discussion

Observation of the *Pdf^01^* mutant, which lacks all PDF expression, has allowed for the identification of PDF's various roles in the control of *Drosophila*'s circadian behavior (e.g. [17], [26], [18]). But the heterogeneous nature of PDF neurons suggests that the various PDF neuron classes likely differ in the extent of their contribution to timekeeping. Therefore, as a first step toward understanding the neuroanatomical basis for PDF's behavioral functions, we asked how PDF from the four classes of PDF neuron might contribute differentially to PDF's first-described roles in the control of locomotor rhythms: maintenance of free-running locomotor rhythms, free-running τ, morning anticipation, and the phase of the evening peak [17].

The l- and s-vLNs are the only PDF neurons that express the molecular clockwork, suggesting that the abdominal PDF cells do not have a direct role in the maintenance of rhythms. However, no previous experiments have addressed the possibility of a circadian function for PDF released from the l- and s-Ab cells. Here we have tested for the necessity and sufficiency of PDF in the abdominal PDF cells by targeted RNAi knockdown of PDF peptide in either the abdominal cells or the vLNs. Our results indicated that normal PDF levels in the l- and s-Ab neurons are neither necessary nor sufficient for PDF's various roles in the maintenance of locomotor rhythms. Thus, PDF from the vLNs must serve these functions. Indeed, knockdown of PDF in the vLNs resulted in the behavioral phenotypes originally described for the *Pdf^01^* mutant [17].

Surprisingly, knockdown of l-vLN PDF had no discernable effects on locomotor rhythms under LD 12∶12 or DD conditions. Thus PDF was not required in the l-vLNs for the maintenance or normal free-running τ under DD conditions or for normal morning anticipation or the phase of the evening peak under LD conditions. To our knowledge, these are the first results to experimentally address the requirement for PDF in the l-vLNs for the normal maintenance of locomotor rhythms. It is important to note that *c929-Gal4*-mediated PDFrnai also had clear effects on PDF levels in the s-Ab cells, but the lack of *Pdf^01^*-like behavioral phenotypes in these flies and in the *Dot-Gal4*-mediated knockdown indicates that PDF is not required in either the l-vLNs or Ab PDF neurons for normal locomotor rhythms under LD 12∶12 or constant conditions.

Knockdown of PDF in the l-vLNs was accompanied by a significant increase in PDF immunoreactivity in the s-vLNs, suggesting that one function of l-vLN PDF is the regulation of PDF levels in the s-vLNs. The s-vLNs are well situated within the aMe to receive l-vLN input [36] and recent work has established that s-vLNs respond to PDF with increases in cAMP [22]. Thus there is both anatomical and physiological evidence to suggest that the s-vLNs are targets of l-vLN PDF. The increase in s-vLN PDF IR that accompanied l-vLN PDF knockdown is further evidence of modulation of the s-vLNs by the l-vLNs.

The s-vLNs are unique among the PDF-positive vLNs in their ability to maintain a rhythm of PERIOD/TIMELESS expression and nuclear entry under constant darkness and temperature [32], [33], suggesting that PDF's role in the maintenance of free-running locomotor rhythms likely emanates from these neurons. Furthermore, the projection patterns of the l- and s-vLNs differ dramatically. For example, the projections of the s-vLNs enervate central brain regions occupied by the clock neurons hypothesized to drive the evening peak of activity (Reviewed in [35]), suggesting that PDF's effects on evening peak phase likely emanate from the s-vLNs. In contrast, the l-vLNs send projections across the ipsi- and contra-lateral optic lobes, and cross the brain via the posterior optic tract, where they may interact with projections from the s-vLNs within the accessory medulla (aMe), a neuropil critical for time-keeping in other insects (e.g., [37]).

Given the fact that PDF expressed in the vLNs accounted for PDF's originally described circadian functions and that specific l-vLN knockdown of PDF had no clear effects on locomotor behavior, we expected that knockdown of s-vLN PDF would behaviorally phenocopy the *Pdf^01^* mutant. Unfortunately, there is currently no Gal4 element that drives expression within the s-vLNs without driving expression within a subset of the l-vLNs. For this reason a conservative interpretation of our results must fall short of a completely clean parsing of PDF functions between the two classes of vLN. Nevertheless, we feel that several observations suggest a specific role for s-vLN PDF in the control of morning anticipation and the maintenance of DD rhythmicity.

Our initial attempt at s-vLN PDF knockdown using the *R6-Gal4* element resulted in only a partial reduction of PDF IR in the soma (63%) and spared PDF in the dorsal projections. Despite the partial nature of this knockdown, these flies displayed clearly reduced morning anticipation under LD and a very high incidence of arhythmicity under DD conditions. Importantly, these phenotypes were accompanied by no measurable loss of PDF IR from the l-vLNs. Therefore, morning anticipation and free-running rhythmicity appeared to be sensitive to PDF levels in the s-vLNs. We wondered if the wild type τ and evening peak phase displayed by these flies was due to the residual PDF in the s-vLNs of *R6-Gal4* knockdown flies, or if PDF from the l-vLNs was sufficient for the maintenance of these features. *Mai179-Gal4*-mediated PDF knockdown resulted in a much greater loss of PDF in the s-vLNs - including an apparently complete knockdown of PDF in the dorsal projections – but also resulted in a partial knockdown of PDF in the l-vLNs. Despite the much greater loss of PDF from the s-vLNs in these flies, and the fact that l-vLN PDF was measurably reduced, τ and evening peak phase were normal. Thus, PDF from the remaining PDF-positive l-vLNs was likely sufficient for the maintenance of normal τ and evening peak phase in these flies.

As for *R6-Gal4*, *Mai179-Gal4*-mediated PDF knockdown resulted in high DD arhythmicity. But unlike the s-vLN PDF knockdown by *R6-Gal4*, *Mai179-Gal4*-mediated knockdown resulted in the loss of PDF IR in the dorsal projections of the s-vLNs. The presence of this PDF projection was previously implicated as critical for the maintenance of free-running locomotor rhythms. Indeed, a single dorsal projection from only one s-vLN appeared to be sufficient for such rhythms [29]. Together with the fact that free-running rhythms are not affected by a loss of PDF from the l-vLNs or Ab PDF neurons, these results make a strong case for the hypothesis that PDF's role in the maintenance of free-running locomotor rhythms emanates solely from the s-vLNs.

The anatomical basis of PDF's role in morning anticipation is less clear. The partial knockdown of s-vLN PDF using *R6-Gal4* resulted in a significant reduction in morning anticipation without measurable effects on PDF expression in the l-vLNs. This suggested that PDF from the s-vLNs was particularly important for such anticipation. The more complete PDF knockdown in the s-vLNs by *Mai179-Gal4* was also accompanied by a measurable reduction in morning anticipation, but this reduction was not significantly different than one of the relevant control lines, casting doubt on the link between s-vLN PDF levels and morning anticipation. It is interesting to note, however, that the morning anticipation phenotype of *Mai179-Gal4* knockdown flies was less severe than that of *R6-Gal4* despite the additional loss of PDF from the l-vLNs. We therefore hypothesize that PDF's role in morning anticipation emanates from the s-vLNs alone, but acknowledge that this hypothesis requires further investigation.

Given the fact that the l-vLNs lack a free-running molecular rhythm and seem anatomically isolated from most non-PDF-expressing clock neurons, it was surprising that PDF from these cells was sufficient for normal τ and evening peak phase in flies with severely reduced PDF in the s-vLNs. It is possible that PDF from the l-vLN might affect τ and evening phase through the s-vLNs, even when the s-vLNs lack PDF. Recent work has indicated that, in addition to PDF, the s-vLNs express short neuropeptide F (sNPF; [38]). Thus, PDF from the l-vLNs might control evening peak and τ by modulating sNPF release from the s-vLNs, which could, in turn, affect the dorsal neurons and dorso-lateral neurons in the dorsal protocerebrum, neurons important for the evening peak of activity (Reviewed in [7], [39]).

A major caveat of RNAi is off-target effects, particularly when Gal4 drivers are expressed in large numbers of non-target neurons, as was the case for several of the lines used here (Supplemental Figure S2). Though we were careful to correlate the behavioral effects of our knockdown experiments with observation and measures of PDF immunoreactivity in PDF neuron subclasses, it is still possible that some effects were due to off-target knockdown of other peptides. The *uas-PDFrnai* construct created by Dietzl et al. [30] is predicted to have zero off-target genes (http://stockcenter.vdrc.at/) based on the predicted sequence of all possible 19-mers produced by the diced double-stranded PDFrnai hairpin [30]. We therefore think it is unlikely that the behavioral effects described here were due to off-target knockdown of other genes.

**Figure 13:**
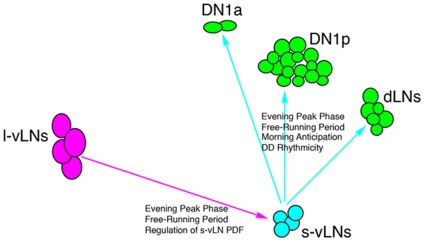
A model of the circadian roles of PDF released from the l- and s-vLN within the clock neuron network. PDF from either class of vLN appears to be sufficient for most of this peptide's circadian functions, though there is some evidence for specialized roles for PDF released from the s-vLNs. We hypothesize that PDF's roles in the maintenance of DD rhythmicity an in morning anticipation under LD emanate predominantly from the s-vLNs. PDF released from the l- or the s-vLNs appears to be sufficient to set τ and evening peak phase. PDF released from the l-vLNs is hypothesized to modulate PDF levels in the s-vLNs, a result consistent with previous work describing anatomical and physiological evidence of modulation of the s-vLNs by the l-vLNs [36], [22].

The results presented here indicate that PDF's roles in circadian locomotion are required to differing extents within the four classes of PDF-expressing neurons (Figure 13). As Normal PDF levels were not required in the l- and s-Ab PDF neurons for normal locomotor rhythms under LD 12∶12 and DD conditions. Surprisingly, loss of PDF from the l-vLNs resulted in no obvious *Pdf^01^*-like behavioral phenotypes under LD or DD conditions. Nevertheless, PDF from the l-vLNs did appear to be sufficient for the setting of evening peak phase under LD and for the maintenance of normal τ under DD conditions. Thus PDF from either class of vLNs was sufficient for normal τ and evening peak phase. Morning anticipation under LD conditions and the persistence of locomotor rhythms under DD were sensitive to the levels of PDF in the s-vLNs and PDF was not required in the l-vLNs for these aspects of rhythmic locomotion. Though the interpretation of these results must be tempered somewhat by a lack of Gal4 drivers that are truly specific to the s-vLNs, they do refine our understanding of the neuroanatomical basis of PDF's various circadian functions (Figure 13). Furthermore, the methods introduced here for the targeted knockdown of PDF can now be extended to the recently established roles of PDF in the control of sleep, arousal, photoperiodic adjustment of locomotor pattern, and geotaxis.

## Methods

### Fly Rearing, Stocks, and Crosses

*Drosophila* were reared on cornmeal/agar media supplemented with yeast and kept in a 25°C incubator with a glass door allowing exposure to the lab's ambient light. The various Gal4 lines used here have all been described previously and were all second chromosome insertions. We used *tim(uas)-Gal4* as our *tim-gal4* [40]. *Dot-Gal4* was first used by Kimbrell and co-workers [41] and chosen based on its recapitulation of ODD-SKIPPED expression (Jim Skeath, personal communication). *c929-Gal4* was first described by Hewes *et al.* [42], *R6-Gal4* by Helfrich-Förster *et al.* [36], and *Mai179-Gal4* by Siegmund and Korge [43]. The *R6-Gal4* and *Mai179-Gal4* elements are homozygous lethal and where therefore maintained over the *CyO* balancer. We obtained the *uas-LacZ^nls^* line from the Blooming Stock Center (stock # 3955). Gal4-driven β-galactosidase expression was determined for male progeny of single crosses of the various Gal4 lines and the *uas-LacZ^nls^* line.

The *yw,uas-dicer2;;* line (*uas-dcr2*) was kindly provided by Stephan Thor at Linköping University. The co-expression of *dcr2*, a gene important for the production of small inhibitory RNAs from double-stranded RNA and the assembly of RNAi silencing complexes in *Drosophila* [44], increases the efficacy of RNAi-mediated knockdown in the fly [30]. The *;;uas-PDFrnai* line was created by Dietzl and coworkers [30] and obtained from the Vienna Drosophila RNAi Center (transformant ID: 4380). For each Gal4 line, we created stable lines with *uas-dcr2* on the X chromosome and the Gal4 element on the second chromosome, either as homozygous lines or with the Gal4 element balanced over *CyO*. For most knockdown experiments *uas-dcr2;Gal4* virgins were crossed to *;;uas-PDFrnai* or *w^1118^* males and *uas-dcr2/y;gal4/+;uas-PDFrnai/+* and *uas-dcr2/y;gal4/+;* male progeny were compared. These genotypes were also compared to *uas-dcr2/y;;uas-PDFrnai/+* males that were the product of a cross between *uas-dcr2;;* virgins and *;;uas-PDFrnai* males.

### Behavioral Recording and Analysis

Locomotor activity of individual flies was recorded using the Drosophila Activity Monitoring (DAM) system (Trikinetics, Waltham, MA) and has been described previously[45], [17]. Briefly, individual males flies were aspirated into Pyrex glass tubes containing a sugar/agarose food source at one end and capped with a cotton plug at the other and placed into DAM monitors. Monitors were placed in 25°C incubators under a single 20W fluorescent bulb (∼1000 lux) and entrained for at least 6 full days under light/dark (LD) cycles of 12 hours of light and 12 hours of darkness, followed by 9 days under constant darkness (DD). The DAM system recorded the number of infrared beam crossing per 30 minutes for each fly. We used χ^2^ periodogram analysis [46] to determine if individual flies were rhythmic under DD. We considered flies with power values of less than 10 to be arrhythmic if width values were also less than 2 to be arrhythmic [47]. Flies with a τ of less than 18 or more than 30 hours were also considered arrhythmic.

To describe the pattern of locomotion under LD 12∶12 conditions we created population “eduction” plots of LD behavior (Supplemental Figures S1 and S3) using the Brandeis Rhythm Package, originally written by David Wheeler and modified by Yiing Lin. These histograms display the pooled proportion of beam crossings that occurred in each 30-minute bin over 6 days of LD entrainment for all the flies of a given genotype in a given experiment. We quantified the extent of morning anticipation using the Anticipation Phase Score (APS) of Harrisingh et al. [48], defined as the percentage of activity in the 6 h period before lights-on that occurs in the 3 hours just before the transition.

Quantification of APS readily detected the difference in morning anticipation between *Pdf^01^* mutants and wild-type controls (Figure 1), but often did not reflect the differing profiles of evening anticipation apparent in the eduction plots. We therefore used the phase of the evening activity peak as a measure of evening anticipation. Using the Brandeis Rhythm package, we determined the peak of evening activity for each fly by smoothing the time series of beam crossings with a two pole Butterworth filter to remove noise [49] and then determined the peak value of the smoothed curve within a 10-hour window centered on ZT 10 (i.e., two hours before lights-off). For each fly, an average phase value for all six days under LD 12∶12 was determined. Comparison of these evening peak phase values readily detected the differing profile of evening peak activity observed for *Pdf^01^* mutants and controls (Table 1). We additionally describe the evening activity of flies by plotting average unfiltered beam crossing counts for the last six hours of the day (ZT6 to ZT12), thereby visualizing both the timing and levels of activity leading up to the lights-off transition.

For most experiments, APS, evening peak phase, τ, and power values were compared by one-way ANOVA followed by a *post-hoc* Tukey's test for all pairs of genotypes. For *Pdf^01^* (w15) mutants, these values were compared to the control w33 line by Student's t-test. All statistical tests were performed using Prism 5 for Mac OSX (Graph Pad, San Diego, CA). All genotypes reported here were behaviorally tested in at least two (typically four) independent runs.

### Immunocytochemistry

The dissection, fixation, and immunocytochemical visualization of PDF, PER, β-galactosidase, and ELAV were as described in Shafer *et al.* [11]. Guinea pig anti-proPDF (PAP-59-IV; [17]) was used at a dilution of 1∶1000. Mouse anti-ELAV (mAb Elav-9F8A9; [50]) was used at a dilution of 1∶10 and was acquired from the Developmental Studies Hybridoma Bank (Iowa City, IA). Rat anti-PERIOD (provided by M. Rosbash) was diluted 1∶500. Mouse anti-β-galactosidase was purchased from Promega (Madison, WI, Cat. #Z3781, Lot #149211) and used at a dilution of 1∶1000. All primary anti-sera were diluted in PBS with 0.3% Triton X-100 (PBS-TX). Alexa-Fluor (488, 568, and 633) conjugated secondary antisera (all raised in goat) were from Molecular Probes (Invitrogen, Carlsbad, CA) and were diluted 1∶1000 in PBS-TX. When comparing PDF Immuno-reactivity levels between genotypes, care was taken to insure that the immunocytochemistry was conducted in parallel using the same reagents, aliquots of antisera, and exposure and rinse times. No quantitative comparisons were made between tissues processed at different times or with different aliquots of sera. Immunocytochemically labeled brains were arranged on poly-lyseine-coated coverslips, dehydrated in a graded glycerol series, and mounted in Hard-Set Vectashield (Vector Laboratories, Burlin game, CA) as described in Shafer *et al.* [11].

### Microscopy

We performed scanning laser confocal microscopy with an Olympus FV500 microscope, using Fluoview acquisition software (Olympus, Center Valley, PA). When comparing whole-CNS PDF expression, PMT voltage, offset, and laser power settings were determined for a control line and maintained for the experimental genotype and any remaining control lines. Gain was always maintained at 1.0x. For whole-CNS montages, we used an XLUMPlanFl 20×/0.95 water immersion lens. At this magnification two overlapping Z-series, one for the brain and one for the ventral nerve cord, were constructed. For the comparison of whole CNS PDF expression between genotypes, Z-steps were 1 µm and the depth of the Z-series was determined by anti-ELAV ICC to insure that comparable volumes were sampled for all genotypes. For the pattern of Gal4-driven β-Galactosidase expression, Z-series depths were determined by anti-β-galactosidase ICC. Z-steps were 1 µm. Z-series projections were created using Image J (available from the NIH at rsb.info.nih.gov/ij/index.html), saved as RGB images, and assembled into montages in Adobe Photoshop (Adobe, San Jose, CA).

The expression of nuclear β-galactosidase in the four classes PDF neuron in flies with various *Gal4/uas-LacZ^nls^* combinations was imaged using a LUMPL 60×/1.10 water objective with correction collar. A digital zoom of 2.5x was used for optical sections of PDF neurons. Anti-PDF immunostaining was visualized using Alexa-Fluor 488; Alexa-Fluor 568 was used for Anti-β-galactosidase. Optical sections were chosen based on PDF expression and sequential scans of the argon and krypton lasers was used to avoid bleed-through.

To compare somatic PDF expression in the vLNs of various genotypes, we imaged single optical sections through individual neurons using the LUMPL 60×/1.10 objective with a 2.5x digital zoom. Optical sections were chosen for each vLN based on Anti-PERIOD ICC in brains dissected between zeitgeber time 22 and 24, times when PERIOD expression is predominantly nuclear in the vLNs [33]. For quantitative anti-PDF ICC in PDFrnai knockdown experiments, Anti-PERIOD immunostaining was visualized using Alexa-Fluor 488 and Alexa-Fluor 568 was used for Anti-PDF. We used sequential scans to avoid bleed-through. The mean pixel intensity of cytoplasmic PDF was quantified using Image J, with the cytoplasmic region of interest determined by PERIOD expression. Mean background pixel intensity was also measured in a region surrounding each neuron and this value was subtracted from each cytoplasmic value. For each genotype, the average, background-subtracted mean pixel intensity of PDF immuno-reactivity (IR) was calculated from five brains for each genotype, with four l- and s-vLNs measured in each brain. Different settings were used for the l- and s-vLNs owing to the weaker PDF immunosignals of the s-vLNs, and the settings for these neurons were held constant between genotypes during each imaging session. For comparisons between genotypes all neurons of a particular class were imaged for each genotype during the same imaging session, always after lasers had been ignited for at least 30 minutes. PDF IR levels were compared using one-way ANOVA followed by a Tukey's test for experiments involving more than two genotypes. Comparisons of pairs of genotypes were done by a Student's t-test.

We used the LUMPL 60×/1.10 water objective to create Z-series reconstructions of PDF expression in the posterior optic tract and dorsal projection in the central brain and in the abdominal PDF neurons in the ventral nerve cord. Anti-ELAV ICC was used to ensure that the same brain regions were sampled for all genotypes. The representative Z-series images presented here reflected the PDF expression observed in at least 10 brains per genotype.

## Supporting Information

Figure S1 Summary of locomotor behavior under LD 12:12 for Pdf01 mutants and uas/gal4 control lines. Each panel displays population averages 6 days of activity for a single genotype. Activity is shown as the proportion of daily beam crossings that occurred within each 30 minute bin. Each plot represents the activity of ∼30 male flies. A. w33 (wild-type control for w15). B. w15 (Pdf01 mutant). C. w1118 (a common white-eyed lab stock). D. y w,uas-Dicer2;; E.;;uas-PDFrnai/+ F.;tim(uas)-Gal4/+; G.;Dot-Gal/+; H.;c929-Gal4/+; I.;R6-Gal4/+; J.;Mai179-Gal4/+;.
(7.57 MB TIF)

Figure S2 Whole CNS expression of Gal4 lines used for PDFrnai knockdown. Each panel shows a representative whole CNS z-series montage of Gal4-driven β-galactosidase (β-gal) expression. For each Gal4 line β-gal is shown singly in black and white (left side of panel) and as a merged color image with PDF (right side of panel). PDF expression is green and β-gal is magenta for all merged images. A. tim-Gal4-driven β-gal expression. E-F B. Dot-Gal4-driven β-gal expression. C. c929-Gal4-driven β-gal expression. D. R6-Gal4-driven β-gal expression. E. Mai179-Gal4-driven β-gal expression.
(7.91 MB TIF)

Figure S3 Summary of locomotor behavior under LD 12:12 for RNAi mediated knockdown of PDF in neuronal subsets and controls. Each panel displays population averages of activity for 6 days for a single genotype. Activity is shown as the proportion of daily beam crossings that occurred within each 30 minute bin. Each plot represents the activity of ∼30 male flies. A. y w,uas-Dicer2;;uas-PDFrnai/+ B. y w,uas-Dicer2;tim(uas)-Gal4/+; C. y w,uas-Dicer2;tim(uas)-Gal4/+;uas-PDFrnai/+ D. y w,uas-Dicer2;Dot-Gal4/+; E. y w,uas-Dicer2;Dot-Gal4/+;uas-PDFrnai/+ F. y w,uas-Dicer2;c929-Gal4/+; G. y w,uas-Dicer2;c929-Gal4/+;uas-PDFrna/+ H. y w,uas-Dicer2;R6-Gal4/+; I. y w,uas-Dicer2;R6-Gal4/+;uas-PDFrnai/+ J. y w,uas-Dicer2;Mai179-Gal4/+ K. y w,uas-Dicer2;Mai179-Gal4/+;uas-PDFrnai/+.
(6.76 MB TIF)

Figure S4 Knockdown of abdominal PDF expression by c929-Gal4 and Dot-Gal4-driven uas-Dcr2 and uas-PDFrnai. Panels on left show a Z-series reconstruction PDF expression in a representative abdominal ganglion. Panels on the right show merged images of PDF (green) and ELAV (blue). A. Representative confocal Z-series reconstruction of PDF expression within the abdominal ganglion of a y w, uas-Dcr2;Dot-Gal4/+; control brain. s-Ab neurons are marked with asterisks. B. Representative confocal Z-series reconstruction of PDF expression with the abdominal ganglion of a y w, uas-Dcr2;Dot-Gal4/+;uas-PDFrnai/+ brain. These ganglia displayed extremely low PDF IR. Confocal settings were identical for A and B. C. Representative confocal Z-series reconstruction of PDF expression within the abdominal ganglion of a y w, uas-Dcr2;c929-Gal4/+; control brain. s-Ab neurons are indicated by asterisks. D. Representative confocal Z-series reconstruction of PDF expression with the abdominal ganglion of a y w, uas-Dcr2;c929-Gal4/+;uas-PDFrnai/+ brain. These ganglia typically lacked PDF IR in the s-Ab neurons. Confocal settings were identical for C and D.
(9.11 MB TIF)
