# Supplementary figures and images for: RNA-Interference Knockdown of Drosophila Pigment Dispersing Factor in Neuronal Subsets: The Anatomical Basis of a Neuropeptide's Circadian Functions
Source: PLoS One. 2009 Dec 14;4(12):e8298. doi: 10.1371/journal.pone.0008298 (PMC2788783; doi:10.1371/journal.pone.0008298)

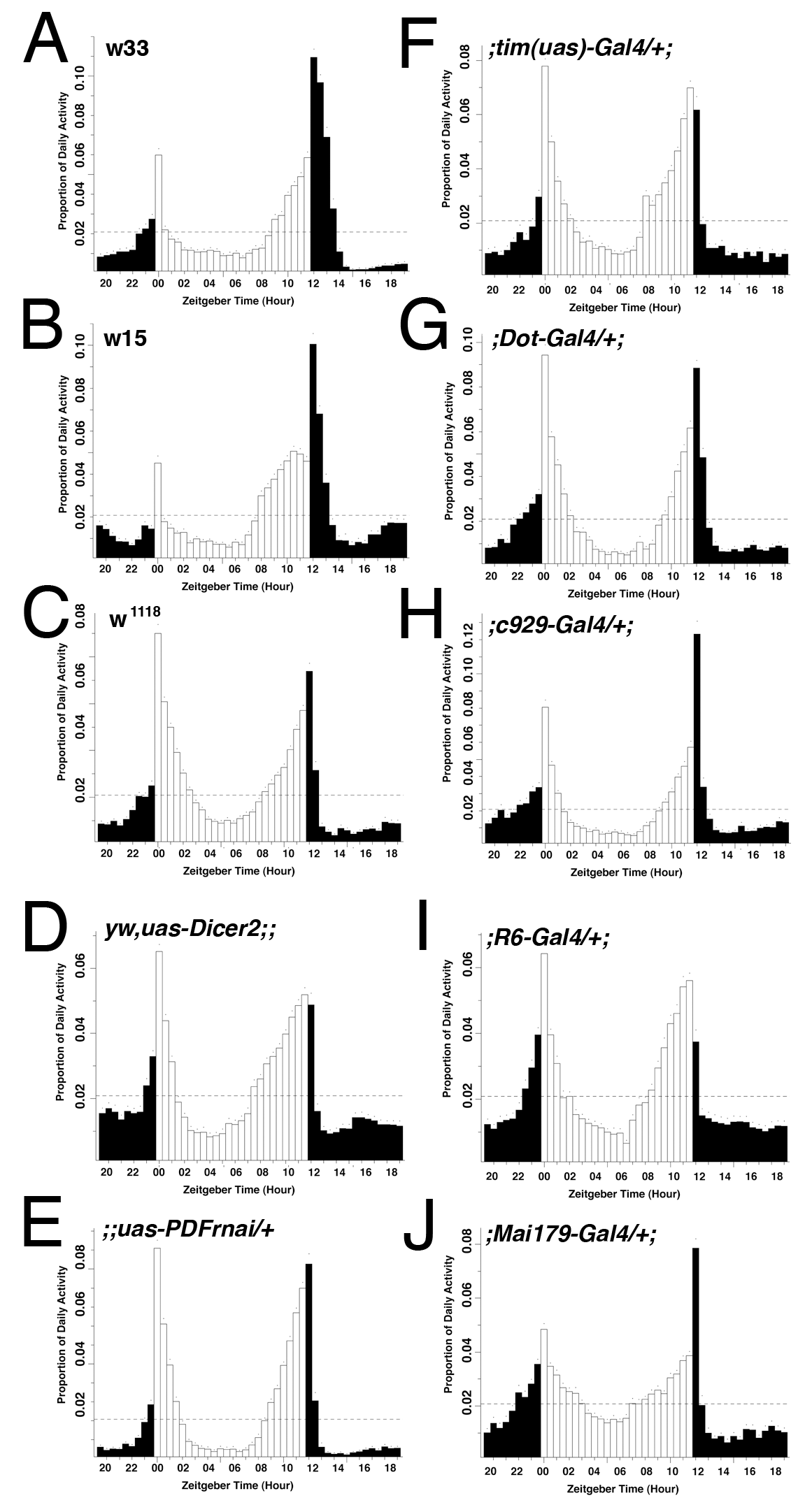

Supplement: Figure S1 — Summary of locomotor behavior under LD 12:12 for Pdf01 mutants and uas/gal4 control lines. Each panel displays population averages 6 days of activity for a single genotype. Activity is shown as the proportion of daily beam crossings that occurred within each 30 minute bin. Each plot represents the activity of ∼30 male flies. A. w33 (wild-type control for w15). B. w15 (Pdf01 mutant). C. w1118 (a common white-eyed lab stock). D. y w,uas-Dicer2;; E.;;uas-PDFrnai/+ F.;tim(uas)-Gal4/+; G.;Dot-Gal/+; H.;c929-Gal4/+; I.;R6-Gal4/+; J.;Mai179-Gal4/+;. (7.57 MB TIF) [file pone.0008298.s001.tif]

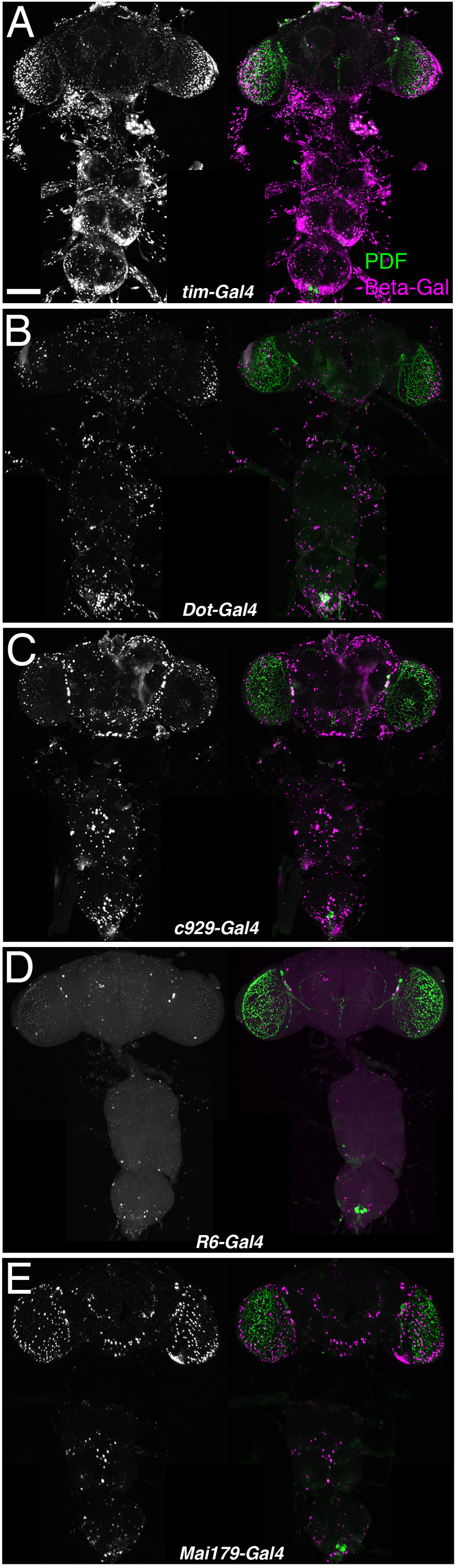

Supplement: Figure S2 — Whole CNS expression of Gal4 lines used for PDFrnai knockdown. Each panel shows a representative whole CNS z-series montage of Gal4-driven β-galactosidase (β-gal) expression. For each Gal4 line β-gal is shown singly in black and white (left side of panel) and as a merged color image with PDF (right side of panel). PDF expression is green and β-gal is magenta for all merged images. A. tim-Gal4-driven β-gal expression. E-F B. Dot-Gal4-driven β-gal expression. C. c929-Gal4-driven β-gal expression. D. R6-Gal4-driven β-gal expression. E. Mai179-Gal4-driven β-gal expression. (7.91 MB TIF) [file pone.0008298.s002.tif]

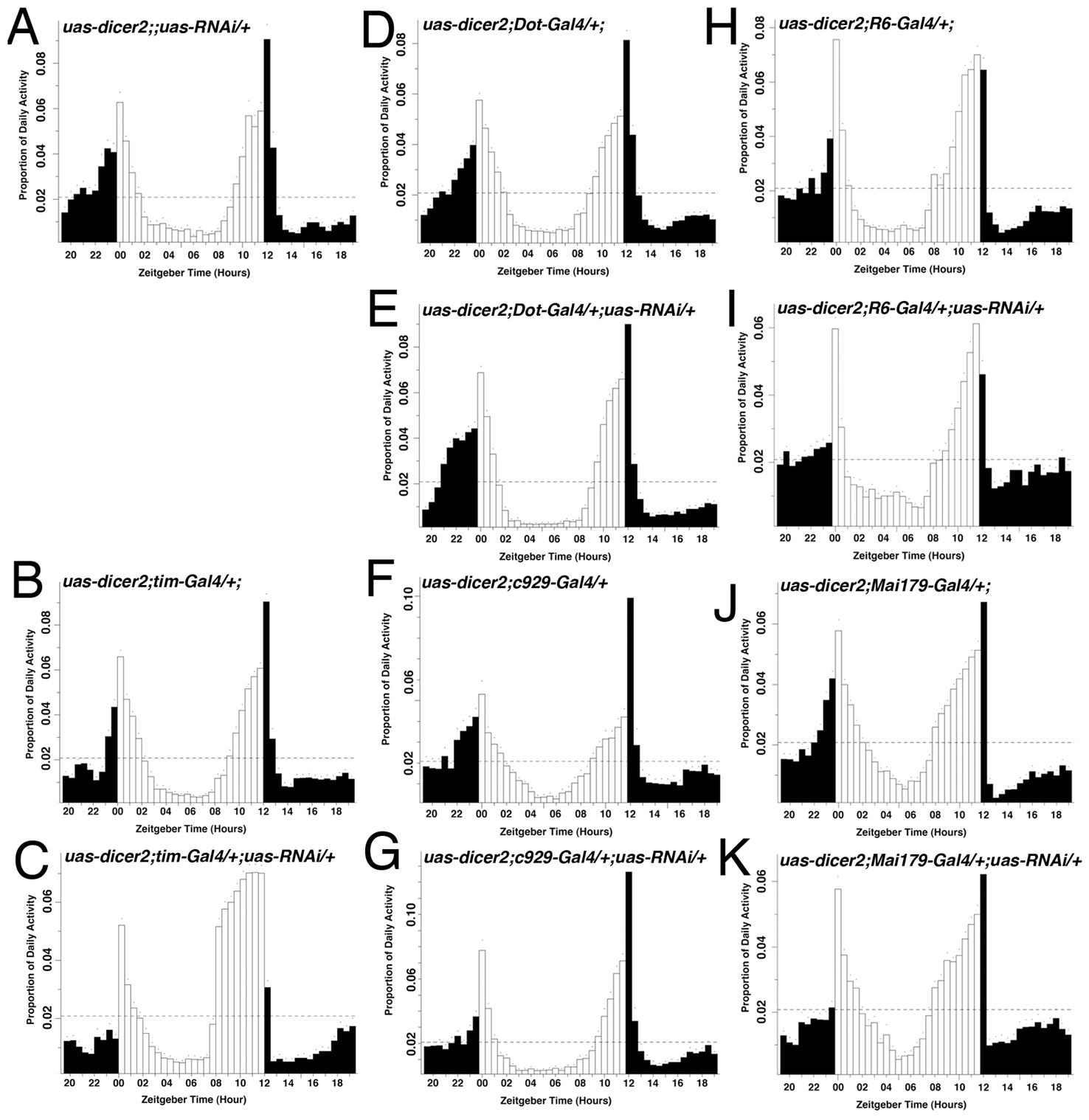

Supplement: Figure S3 — Summary of locomotor behavior under LD 12:12 for RNAi mediated knockdown of PDF in neuronal subsets and controls. Each panel displays population averages of activity for 6 days for a single genotype. Activity is shown as the proportion of daily beam crossings that occurred within each 30 minute bin. Each plot represents the activity of ∼30 male flies. A. y w,uas-Dicer2;;uas-PDFrnai/+ B. y w,uas-Dicer2;tim(uas)-Gal4/+; C. y w,uas-Dicer2;tim(uas)-Gal4/+;uas-PDFrnai/+ D. y w,uas-Dicer2;Dot-Gal4/+; E. y w,uas-Dicer2;Dot-Gal4/+;uas-PDFrnai/+ F. y w,uas-Dicer2;c929-Gal4/+; G. y w,uas-Dicer2;c929-Gal4/+;uas-PDFrna/+ H. y w,uas-Dicer2;R6-Gal4/+; I. y w,uas-Dicer2;R6-Gal4/+;uas-PDFrnai/+ J. y w,uas-Dicer2;Mai179-Gal4/+ K. y w,uas-Dicer2;Mai179-Gal4/+;uas-PDFrnai/+. (6.76 MB TIF) [file pone.0008298.s003.tif]

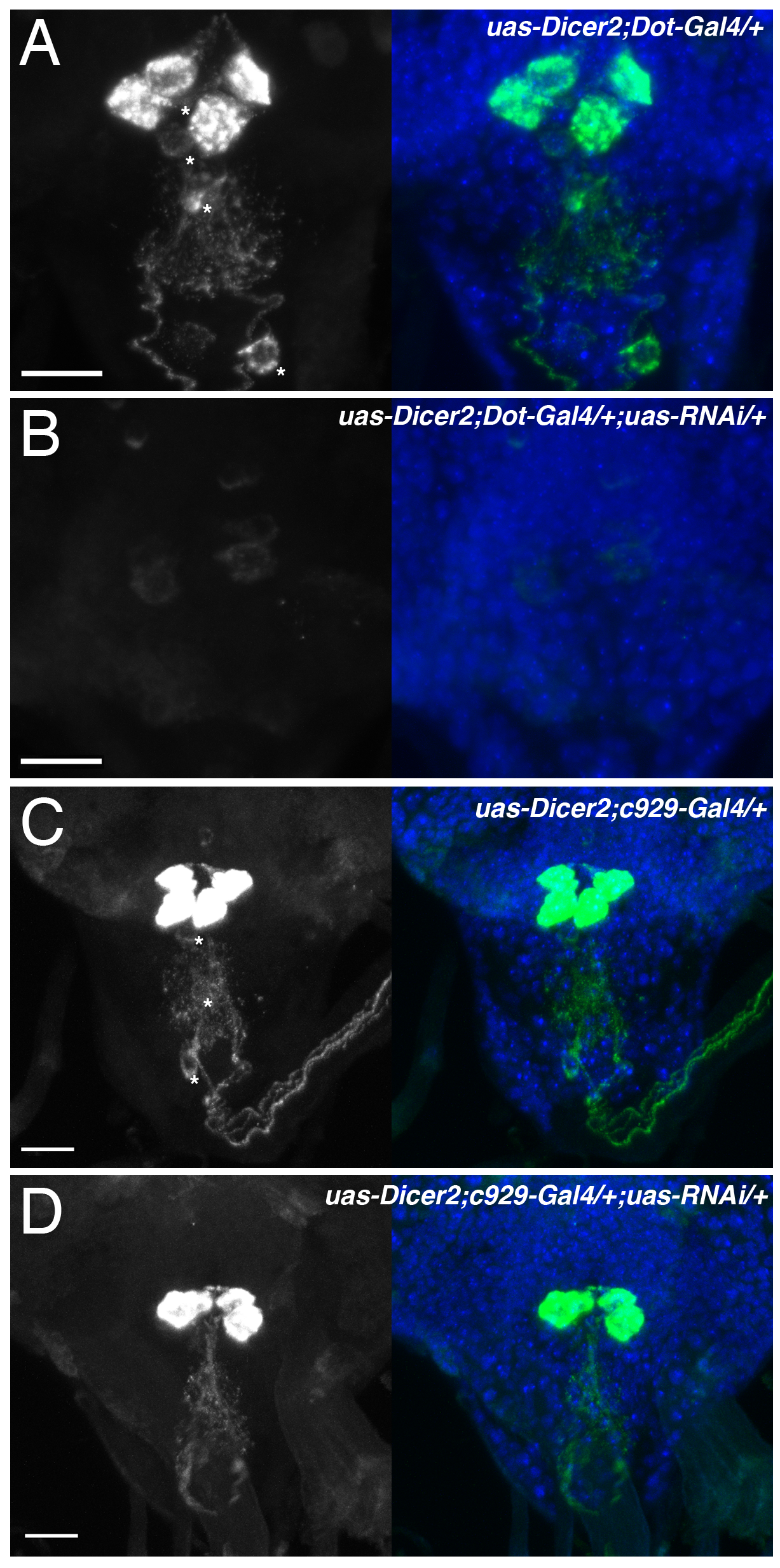

Supplement: Figure S4 — Knockdown of abdominal PDF expression by c929-Gal4 and Dot-Gal4-driven uas-Dcr2 and uas-PDFrnai. Panels on left show a Z-series reconstruction PDF expression in a representative abdominal ganglion. Panels on the right show merged images of PDF (green) and ELAV (blue). A. Representative confocal Z-series reconstruction of PDF expression within the abdominal ganglion of a y w, uas-Dcr2;Dot-Gal4/+; control brain. s-Ab neurons are marked with asterisks. B. Representative confocal Z-series reconstruction of PDF expression with the abdominal ganglion of a y w, uas-Dcr2;Dot-Gal4/+;uas-PDFrnai/+ brain. These ganglia displayed extremely low PDF IR. Confocal settings were identical for A and B. C. Representative confocal Z-series reconstruction of PDF expression within the abdominal ganglion of a y w, uas-Dcr2;c929-Gal4/+; control brain. s-Ab neurons are indicated by asterisks. D. Representative confocal Z-series reconstruction of PDF expression with the abdominal ganglion of a y w, uas-Dcr2;c929-Gal4/+;uas-PDFrnai/+ brain. These ganglia typically lacked PDF IR in the s-Ab neurons. Confocal settings were identical for C and D. (9.11 MB TIF) [file pone.0008298.s004.tif]
